# Comparative genomics provides insights into the potential biocontrol mechanism of two *Lysobacter enzymogenes* strains with distinct antagonistic activities

**DOI:** 10.3389/fmicb.2022.966986

**Published:** 2022-08-11

**Authors:** Shuai Xu, Ziyu Zhang, Xuewen Xie, Yanxia Shi, Ali Chai, Tengfei Fan, Baoju Li, Lei Li

**Affiliations:** Institute of Vegetables and Flowers, Chinese Academy of Agricultural Sciences, Beijing, China

**Keywords:** *Lysobacter enzymogenes*, comparative genomic analysis, secondary metabolites, antagonistic activity, bacterial secretory system, biological control

## Abstract

*Lysobacter enzymogenes* has been applied as an abundant beneficial microorganism to control plant disease; however, most *L. enzymogenes* strains have been mainly reported to control fungal diseases, not bacterial diseases. In this study, two *L. enzymogenes* strains were characterized, of which CX03 displayed a broad spectrum of antagonistic activities toward multiple bacteria, while CX06 exhibited a broad spectrum of antagonistic activities toward diverse fungi and oomycete, and the whole genomes of the two strains were sequenced and compared. The genome annotation showed that the CX03 genome comprised a 5,947,018 bp circular chromosome, while strain CX06 comprised a circular 6,206,196 bp chromosome. Phylogenetic analysis revealed that CX03 had a closer genetic relationship with *L. enzymogenes* ATCC29487^T^ and M497-1, while CX06 was highly similar to *L. enzymogenes* C3. Functional gene annotation analyses of the two *L. enzymogenes* strains showed that many genes or gene clusters associated with the biosynthesis of different secondary metabolites were found in strains CX03 and CX06, which may be responsible for the different antagonistic activities against diverse plant pathogens. Moreover, comparative genomic analysis revealed the difference in bacterial secretory systems between *L. enzymogenes* strains CX03 and CX06. In addition, numerous conserved genes related to siderophore biosynthesis, quorum sensing, two-component systems, flagellar biosynthesis and chemotaxis were also identified in the genomes of strains CX03 and CX06. Most reported *L. enzymogenes* strains were proven mainly to suppress fungi, while CX03 exhibited direct inhibitory activities toward plant bacterial pathogens and showed an obvious role in managing bacterial disease. This study provides a novel understanding of the biocontrol mechanisms of *L. enzymogenes*, and reveals great potential for its application in plant disease control.

## Introduction

*Lysobacter* species are widely distributed in nature, including in soil, fresh water, and even some extreme environments (Ramamoorthy et al., 2001; Folman et al., 2003). The *Lysobacter* genus was first proposed in 1978 and was named for its ability to produce multiple antibiotics that dissolve the cell walls or membranes of other microorganisms (Panthee et al., 2016). *Lysobacter* strains are characterized as sliding and glutinous on agar medium plate (de Bruijn et al., 2015). *Lysobacter* belongs to the *Xanthomonadaceae* family of *Gammaproteobacteria*, and was once indistinguishable from *Polyangium* and *Sorangium* or confused with *Xanthomonas* and *Stenotrophomonas*. At the time it was identified as a separate genus, *Lysobacter* contained four species: *L. antibioticus*, *L. brunescens*, *L. enzymogenes*, and *L. gummosus* (Hayward et al., 2010). To date, approximately 25 *Lysobacter* species have been identified, but only a few strains have been subjected to genome-wide. Recently, *Lysobacter* has been described as a new source for bioactive natural products, and has been used to control plant disease. For example, *L. enzymogenes* OH11 produced heat-stable antifungal factor (HSAF) that showed degrading activity against the chitinous hyphae of *Fusarium graminearum* (Odhiambo et al., 2017). A 2,5-diketopiperazine produced by *L. capsica* AZ78 showed significant antagonistic activities toward *Phytophthora infestans* and *Plasmopara viticola* (Puopolo et al., 2014). *L. antibiobioticus* HS124 produced 4-hydroxyphenylacetic acid (4-HPAA), an antibiotic that was able to control the root-knot nematode in tomato (Lee et al., 2013).

*Lysobacter enzymogenes* has been reported as a potential biocontrol agent to control diverse plant diseases due to its capacity to generate secondary metabolites with antibiotic activity or generate a variety of extracellular enzymes with degrading ability toward the cell walls of fungal pathogens (Hayward et al., 2010; Xie et al., 2012). *L. enzymogenes* C3 was reported to inhibit wheat root rot caused by *Bipolaris sorokiniana* (Zhang and Yuen, 1999), lawn brown spot triggered by *Rhizoctonia solani* (Giesler and Yuen, 1998), and soybean rust resulting from *Uromyces appendiculatus* (Yuen et al., 2001). In addition, strain C3 had strong antagonistic activity against *Pythium ultimum* and various nematodes (Kobayashi and Yuen, 2005; Li et al., 2008). *L. enzymogenes* OH11 effectively antagonized the growth of many plant pathogenic fungi, including *R. solani*, *P. capsica* and *P. aphanidermatum* (Qian et al., 2009). *L. enzymogenes* 3.1T8 was isolated from the inter-root of cucumber and showed an antagonistic effect against *P. aphanidermatum* and exhibited a significant effect in controlling *Corynespora cassiicola* and *P. capsica* disease (Folman et al., 2003). *L. enzymogenes* M497-1 was reported to have broad bacteriolytic specificity and was used to lyse anaerobic Gram-positive rods, including *Micrococcus radiodurans*, *Staphylococcus aureus* and *Peptococcus saccharolyticus* (Takami et al., 2017). *L. enzymogenes* N4-7 was used as a biocontrol agent to control plant fungal diseases and nematodes for the production of β-1,3-glucanase, which has hydrolysis activity on fungal cell walls (Palumbo et al., 2003). In addition, *L. enzymogenes* LE16 reduced the organic phosphorus and increased the soluble P in the soil, thus promoting plant growth (Chen et al., 2019). However, most reported *L. enzymogenes* strains possess the ability to suppress plant pathogenic fungi, oomycete or nematodes, while very few *L. enzymogenes* strains have been reported to control bacterial disease.

It was reported that the reason *Lysobacter* spp. can control plant disease involved in their ability to generate a variety of lytic enzymes and antibiotics. Previous studies showed that *Lysobacter* strains harbored large numbers of genes related to antagonistic activities, and whole-genome annotation indicated that each *Lysobacter* strain shared 12–16 secondary metabolites (Panthee et al., 2016). *L. enzymogenes* was proven to suppress fungi with multiple secondary metabolites and lytic antibiotics. The cyclic lipodepsipeptide HSAF derived from *L. enzymogenes* C3 inhibited the activity of fungal neuro phthalamide synthase and altered the composition of sphingolipids in the cell membrane, resulting in depolarization of the target fungal hyphae (hyphae expansion, numerous branches, etc.) and thus exhibited potent antifungal activity (Kobayashi and Yuen, 2005; Yuen et al., 2018). *L. enzymogenes* OH11 could also produce HSAF, which showed significant antagonistic activities toward diverse pathogenic fungi, and was reported to be a highly effective and broad-spectrum antibiotic of microbial origin. HSAF was not toxic to plants or animals, and maintained high antagonistic activity at high temperatures and under acidic or alkaline conditions, demonstrating good potential for development and application (Yu et al., 2007; Olson et al., 2015). In addition, many other functional genes were closely related to the biosynthesis of HSAF. Overexpression of the quorum sensing related gene *lesR* significantly impaired HSAF biosynthesis levels and antifungal activities (Qian et al., 2014). Deletion of *letR* (a TetR-family protein gene) in *L. enzymogenes* OH11 could significantly increase HSAF levels and the transcription of key biosynthetic genes (Wang et al., 2017). Other non-ribosomal peptides produced by *L. enzymogenes* were also reported to have antipathogenic activities. WAP-8294A, a cyclic lipodepsipeptide isolated from *L. enzymogenes* OH11, was reported to have potent activity against Gram-positive bacterial pathogens, especially *S. aureus* (Hashizume et al., 2001). However, most studies have focused on the antifungal compounds produced by *L. enzymogenes*, especially HSAF, while only a few reports of secondary metabolites have been related to antibacterial activity. Previous studies showed that *L. enzymogenes* degraded phytopathogenic (micro)organism cell walls by secreting a variety of highly active extracellular hydrolases, such as protease cellulases, glucanases and chitinases. *L. enzymogenes* strains C3, OH11 and N4–7 synthesized three extracellular β-1,3-glucanases encoded by *glu*ABC genes (Palumbo et al., 2003, 2005). The capacity of the strain to generate β-1,3-glucanases and control brown patches in tall fescue was reduced when all three genes of *L. enzymogenes* C3 were mutated simultaneously (Palumbo et al., 2005). *L. enzymogenes* OH11 secreted proteases that significantly inhibited the growth of *R. solani*. The α-hydrolytic protease secreted by *L. enzymogenes* 3.1T8 could degrade proteins in the nematode body wall, and effectively antagonize nematode-induced plant diseases (Folman et al., 2004). In addition, *L. enzymogenes* OH11 used the type IV secretion system (T4SS) as the main contact dependent weapon to control *Pectobacterium carotovorum* (Shen et al., 2021). Type IV pilus-driven twitching motility, which was proven to promote bacterial colonization on host fungal surfaces in *L. enzymogenes* OH11, was also recognized as one of the antifungal mechanisms (Xia et al., 2018). Moreover, induced resistance was also described as a mechanism for disease control of *L. enzymogenes* strains. Resistance elicited by *L. enzymogenes* C3 suppressed the conidial germination of *B. sorokiniana* on the phylloplane, thus reducing the severity of leaf spot (Kilic-Ekici and Yuen, 2003). In addition, other *Lysobacter* species applied to biological control have similar antagonistic mechanisms. *L. antibioticus* HS124 showed a significant control effect on *Phytophthora* Blight for the generation of 4-hydroxyphenylacetic acid and many lytic enzymes (Ko et al., 2009). Notably, *L. antibioticus* OH13 produced phenazine antibiotics that showed antagonistic activities against several bacteria (Zhao et al., 2016). *L. capsica* AZ78 produced cyclo (_L_-Pro-_L_-Tyr), a 2,5-diketopiperazine that was effective in inhibiting the growth of the sporangia of *P. infestans* (Puopolo et al., 2014).

In this study, two *L. enzymogenes* strains CX03 and CX06 exhibited distinct antagonistic activities toward different plant pathogens. To reveal the possible different biocontrol mechanisms of the two strains, the genomes of CX03 and CX06 were sequenced and compared with those of the typical *Lysobacter* strains C3, M497-1, 55 and 76, which were reported to be beneficial to plant growth. Phylogenetic analysis was used to demonstrate the taxonomic positions of CX03 and CX06, and their relationships with other *Lysobacter* strains. Core genes related to the biosynthesis of known and unknown secondary metabolites of the different *Lysobacter* strains were predicted and compared. Comparative genome analysis was performed to mine the common and unique functional genes involved in other biocontrol factors, such as the bacterial secretion system and the biosynthesis of lyase. These data will provide novel insights into the biocontrol mechanisms of *L. enzymogenes* and provide new antibiotic resources for disease control.

## Materials and methods

### Strain isolation, culture conditions, microscopic analysis and bacterial DNA extraction

Strains CX03 and CX06 were isolated from rhizosphere soil in Guizhou Province and swamp soil in Sichuan Province, China, according to a standard 10-fold dilution plating assay as described by previous study, respectively (Wang et al., 2013). Strains CX03 and CX06 grew stably on nutrient broth (NB) agar medium at 28°C. Gliding motility of strains CX03 and CX06 were evaluated on 5% tryptic soy agar (TSA) medium according to the reported method (Zhou et al., 2015). Strains CX03 and CX06 were inoculated to NB media (1/1000 inoculation proportion) with shaking at 28°C, and the colony number of CX03 and CX06 and the pH value of CX03 and CX06 culture were measured every four hours. The genomic DNA of strains CX03 and CX06 was extracted from the cultured bacterial colonies (OD_600_ = 1.0) according to the manufacturer’s instructions of the TIANamp Bacteria DNA kit (Tiangen Biotech (Beijing, China) Co., Ltd.). Scanning electron microscopy (SEM) and transmission electron microscopy (TEM; SU8010, Hitachi, Japan, 10.0 kV) were used to observe the micromorphology of the two strains.

### Antagonistic and biocontrol assays

The antagonistic abilities of strains CX03 and CX06 against different plant pathogens were measured by plate bioassays, and the control efficacies of strains CX03 and CX06 against cabbage black rot and the stem rot of Chinese cabbage were tested in pot experiments. Briefly, the cabbage was inoculated with *X. campestris* pv. *campestris* (1 × 10^8^cfu/ml) by spray method, 1 day later, the culture of CX03 and CX06 (1 × 10^8^cfu/ml) were inoculated to cabbage (inoculated with *X. campestris* pv. *campestris*) by spray method, respectively, and kasugamycin was used as chemical control. The Chinese cabbage was inoculated with *R. solani* by root irrigation, 1 day later, the culture of CX03 and CX06 (1 × 10^8^cfu/ml) were inoculated to Chinese cabbage (inoculated with *R. solani*), respectively, and validamycin was used as chemical control. Plate tests were used to evaluate the ability to produce siderophores, proteases (Prts), and chitinases according to previously reported methods (Murata, 1991; Chatterjee et al., 1995).

### Biosynthesis of selenium nanoparticles

The selenium (Se) nanoparticle (SeNP) biosynthesis capacities of strains CX03 and CX06 were measured based on previously described method (Zhu et al., 2018) with slight adjustment. The selenium standard curve was plotted according to the previously reported method (Biswas et al., 2011). The activated strains CX03 and CX06 were inoculated in LB medium containing 2.5, 5, 10, 15, 45, and 75 mM Se, and the samples were taken after 24 h of continuous shaking cultivation. The culture was centrifuged at 10,000 rpm for 20 min, the sediment bacteria were resuspended in ddH_2_O, and the above steps were repeated three times. One milliliter of suspension was placed in a 4 ml centrifuge tube, and 1 ml of Na_2_S (1 M) solution was added and mixed well for 1 h. Then, the absorbance value at 500 nm was measured by ultraviolet spectrophotometry and each sample was repeated three times. The measured absorbance was used to calculate the concentration of selenium nanoparticles in the samples according to the standard curve.

### Whole-genome sequencing and annotation

The genomes of strains CX03 and CX06 were sequenced using a PacBio Sequel platform by Allwegene Technologies Corporation, Beijing, China. Circular genome visualization of the two strains were created by CGView (Tatusova et al., 2016). The functional genes were predicted using GeneMarkS software (version 4.17). Transfer RNA (tRNA) and ribosomal RNA (rRNA) were predicted through tRNAscan-SE version 2.0 (Lowe and Chan, 2016) and RNAmmer version 1.2 (Lagesen et al., 2007), respectively. Gene Ontology database (GO) (Ashburner et al., 2000), Kyoto Encyclopedia of Genes and Genomes (KEGG) (Kanehisa et al., 2006), RAST (Rapid Annotation using Subsystem Technology) (Aziz et al., 2008), Pfam^1^, Swiss-Prot^2^ (Bairoch and Apweiler, 2000), Carbohydrate-Active enZYmes Database (CAZy) (Cantarel et al., 2009), Transporter Classification Database (TCDB) (Saier et al., 2009) and the enhanced COG database (Bairoch and Apweiler, 2000; Tatusov et al., 2003; El-Gebali et al., 2019).

### Comparative genomic analysis

The taxonomic position of two strains CX03 and CX06 were confirmed by multilocus gene sequence analysis (MLSA) according to the sequences of conserved genes (16S rRNA, *gyr*B, *atp*D and *rpo*D) (Supplementary Table 1). Sequence alignment of strains CX03 and CX06 with other *Lysobacter* strains was performed using MUSCLE and the phylogenetic tree was constructed through the maximum likelihood method in MEGA 6.0 (Tamura et al., 2013). In addition, average nucleotide identity (ANI) and *in silico* DNA–DNA hybridization (DDH) were executed as described previously (Goris et al., 2007). According to the phylogenetic analysis, four representative strains with released complete genomes, including *L. enzymogenes* M497-1 (AP014940.1), *L. enzymogenes* C3 (CP013140.1), *L. capsici* 55 (CP011130.1) and *L. antibioticus* 76 (CP011129.1) were chosen for comparative genomic analysis. Genome pairwise alignment was conducted using MAUVE comparison software, and Venn diagrams were generated using R package (Richter and Rosselló-Móra, 2009).

### Functional genes related secondary metabolites and microbe–plant interactions

Gene clusters involved in the biosynthesis of secondary metabolites were predicted using antiSMASH 2.0^3^, and compared among the *L. enzymogenes* CX03, *L. enzymogenes* CX06, *L. enzymogenes* M497-1, *L. enzymogenes* C3, *L. capsici* 55, and *L. antibioticus* 76 strains. Functional genes involved in the bacterial secretion system, quorum sensing, two-component system and seleno compound metabolism, biosynthesis of siderophores, flagella and chemotaxis, polyketone, lipopolysaccharide, and peptidoglycan biosynthesis were retrieved according to NCBI databases. The homology analysis of sequences for various functional genes in *L. enzymogenes* CX03, *L. enzymogenes* CX06, *L. enzymogenes* M497-1, *L. enzymogenes* C3, *L. capsici* 55 and *L. antibioticus* 76 were compared based on the KEGG database at the amino acid level.

## Results

### Antagonistic activity and biochemical characteristics

#### Antagonistic activity and pot experiment

**FIGURE 1:**
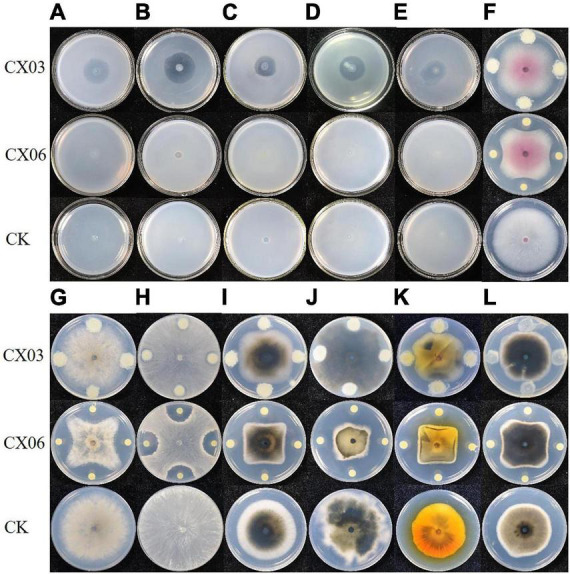
Antagonistic assay of strains CX03 and CX06 against various plant pathogens. **(A)** *Clavibacter michiganensis* subsp. *sepedonicus*, **(B)** *C. michiganensis* subsp. *michiganensis*; **(C)** *Agrobacterium vitis*, **(D)** *Xanthomonas campestris* pv. *campestris*, **(E)** *Pseudomonas syringae* pv. *lachrymans*, **(F)** *Fusarium oxysporum*, **(G)** *Botrytis cinerea*, **(H)** *Rhizoctonia solani*, **(I)** *Corynespora cassiicola*, **(J)** *Diaporthe batatas*, **(K)** *Stemphylium solani*, **(L)** *Phytophthora capsica*. CK, control.

**FIGURE 2:**
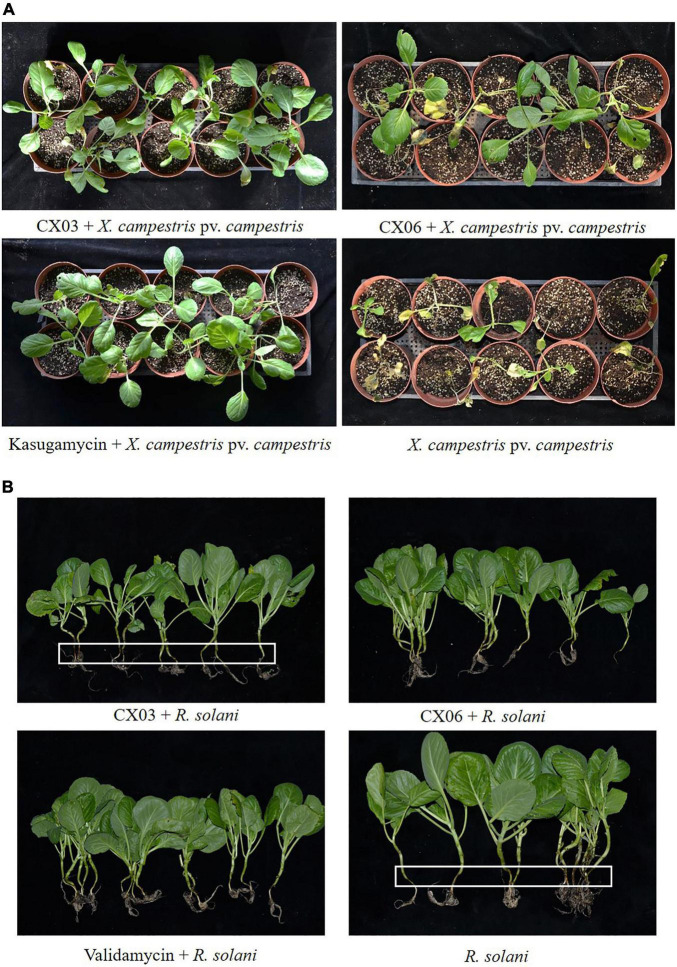
Control efficiency of *L. enzymogenes* CX03 and CX06 against black rot on cabbage **(A)** and stem rot on Chinese cabbage **(B)**. The white frames indicated the pathogenesis of stem rot on Chinese cabbage. Kasugamycin and validamycin were used as chemical control, respectively.

Antagonistic spectrum assays revealed that the two strains CX03 and CX06 displayed the opposite results. Strain CX03 exhibited broad, strong antagonistic activities toward multiple plant-pathogenic bacteria, including *X. campestris* pv. *campestris*, *Clavibacter michiganensis* subsp. *sepedonicum*, *Ralstonia solanacearum*, *Pseudomonas syringae* pv. *tomato*, and *P. syringae* pv. *lachrymans*, and slight inhibitory activities toward plant-pathogenic fungi *F. oxysporum* and *C. cassiicola*, while strain CX06 showed strong inhibitory activities toward many plant-pathogenic fungi including *Verticillium dahlia*, *C. cassiicola*, *Botrytis cinerea*, *F. oxysporum*, *Colletotrichum* spp., *R. solani* and the oomycete *P. capsici* (Figure 1). Furthermore, strain CX03 showed a notable effect in controlling black rot on cabbage (Figure 2), while CX06 exhibited an obvious influence in controlling stem rot on Chinese cabbage (Figure 2). *Lysobacter* sp. was reported to produce siderophores which were associated with the biocontrol mechanisms. Such as *L. enzymogenes* strains C3 and LE16 were all reported to produce siderophores (de Bruijn et al., 2015; Chen et al., 2020). In the study, orange halos appeared around colonies of strains CX03 and CX06 on CAS agar plates, indicating that the two strains produced siderophores (Supplementary Figure 1). *L. enzymogenes* secreted a variety of extracellular hydrolases and were related to its antagonistic activity (Xie et al., 2012). In our study, enzyme activity assays showed that strains CX03 and CX06 produced chitinase (Supplementary Figure 1) and protease (Supplementary Figure 1).

#### Organism information

The colonies of strains CX03 and CX06 were creamy-yellow, with irregular, flat to convex forms with undulated edges (Supplementary Figure 1). Strains CX03 and CX06 were measured as rod-shaped bacteria, and displayed gliding motility (Supplementary Figure 1). Strains CX03 and CX06 could grow in 1% NaCl (w/v), at an optimum temperature 28–30°C, and an optimum pH of 8. CX03 and CX06 were positive for citrate and gelatin hydrolysis tests. Biology assays showed that strain CX03 could utilize D-maltose, D-trehalose, D-fructose, D-galactose, and D-galacturonic acid, while CX06 could utilize D-maltose, gentiobiose, D-melibiose, D-mannose, and L-fucose. The growth curve showed that strains CX03 and CX06 were in the rapid growth stage between 16 and 20 h after inoculation, and reached a stable period at 32 h after incubation (OD_600_ = 2.8) (Supplementary Figure 2A). The pH of the fermentation broth of biocontrol agents may be continuously change during the fermentation. In our study, the pH of strains CX03 and CX06 fermentation rose approximately from 6.5 to 8.3 within 48 h of incubation and tended to be stable (Supplementary Figure 2B).

### Comparison of CX03, CX06 and other sequenced *Lysobacter* strains

#### General genomic features of *Lysobacter enzymogenes* CX03 and CX06

**FIGURE 3:**
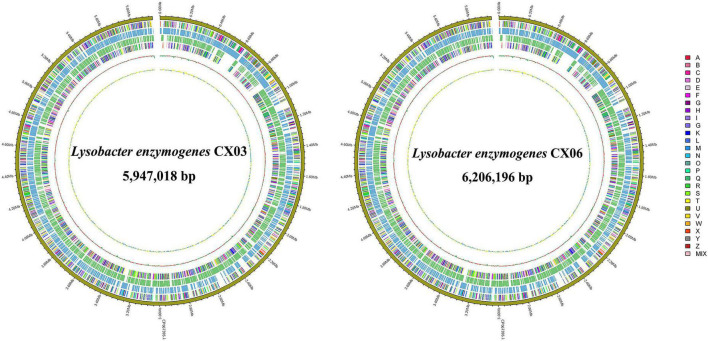
Graphical circular maps of the *L. enzymogenes* CX03 and CX06 chromosome genomes generated using the CGview server. From outside to center, ring 1 showed the genome sequence position, rings 2 and 5 showed protein-coding genes oriented in the forward (colored by COG categories) and reverse (colored by COG categories) directions, respectively. Rings 3 and 4 showed coding genes in the forward (blue) and reverse (green) directions, respectively. Ring 6 showed the G+C% content and the inner most ring showed the GC skews, where blue indicated positive values and yellow indicated negative values.

The complete genome of *L. enzymogenes* CX03 comprised a 5,947,018 bp chromosome with an average G+C content of 69.67%, and the complete genome of *L. enzymogenes* CX06 comprised a circular 6,206,196 bp chromosome with an average G+C content of 69.91% (Supplementary Table 2). The genome structure and functions of strains CX03 and CX06 were shown in the graphical circular genome map (Figure 3). In total, 5,065 genes were predicted in the genome of strain CX03, including 4,957 protein coding genes, 69 RNA genes, and 39 pseudogenes. Using the KEGG, Swiss-Prot, GO, Pfam, NR, TCDB, PHI, and VFDB databases, 4.689 (92.58%), 1,631 (32.20%), 2,958 (58.40%), 2,958 (58.40%), 4,755 (93.88%), 319 (6.30%), 464 (9.20%), and 273 (5.40%) of the ORFs in the CX03 genome were classified into different groups, respectively (Supplementary Table 2). For strain CX06, 5,158 ORFs were predicted in the genome, including 5,059 protein coding genes, 70 RNA genes, and 29 pseudogenes. Similarly, according the KEGG, Swiss-Prot, GO, Pfam, NR, TCDB, PHI, and VFDB databases, 4.867 (94.36%), 1,642 (31.83%), 2,965 (57.48%), 2,965 (57.48%), 4,908 (95.15%), 323 (6.26%), 466 (9.03%), and 328 (6.36%) of the ORFs in CX06 genomes were classified into different groups, respectively (Supplementary Table 2).

Meanwhile, 4,101 functional genes in the CX03 genome and 4,044 protein-coding genes in the CX06 genome were annotated to COG categories, including transcription, cell wall/membrane/envelope biogenesis, amino acid transport and metabolism, lipid transport and metabolism, translation, ribosomal structure and biogenesis (Supplementary Table 3). In addition, 895 genes in the CX03 genome and 1,044 genes in the CX06 genome were not annotated in the COG database, and the functions need to be further verified.

#### Comparison of the CX03 and CX06 genomes with other typical *Lysobacter* strains

Four publicly available typical *Lysobacter* strains with released complete genome sequences, *L. enzymogenes* M497-1, *L. enzymogenes* C3, *L. capsici* 55 and *L. antibioticus* 76 were selected for comparative genomic analysis. The results showed that the whole genome sizes of the six *Lysobacter* strains ranged from 5.92 to 6.39 Mb, with G+C contents of 66.60–69.90%, and the predicted CDSs ranged from 4,801 to 5,198. Moreover, the genome size of strain CX03 was larger than that of strain 76, but smaller than those of strains CX06, M497-1, C3 and 55. In addition, all the six *Lysobacter* strains contained one circular chromosome without a plasmid (Supplementary Table 4).

A phylogenetic tree was constructed according to four conserved genes (16S rRNA, *gyrB*, *atpD* and *rpoB*) to reveal the genetic relationships of *L. enzymogenes* CX03 and CX06 with other representative *Lysobacter* strains. The phylogenetic analysis indicated that the *Lysobacter* species could be clustered into two primary clades. The first clade consisted of three groups including *L. enzymogenes*, *L. capsici*, and *L. antibioticus* species, and strains CX03 and CX06 were clearly classified as *L. enzymogenes* (Supplementary Figure 3). Based on the observed distance relationships, the two strains CX03 and CX06 were clustered in different subclades of *L. enzymogenes*, and *L. enzymogenes* CX03 was closely related to *L. enzymogenes* ATCC 29487^T^, and M497-1, while *L. enzymogenes* CX06 was closely related to *L. enzymogenes* YC36, C3, and OH11.

Average nucleotide identity (ANI) and *in silico* DNA–DNA hybridization (DDH) were used to calculate the similarity of bacteria, and strains exhibiting ANI values ≥96% and DDH values ≥70% were usually regarded as the same species (Zhang et al., 2016). However, low ANI and DDH values were calculated between CX03 and other *Lysobacter* strains, even the *L. enzymogenes* strains (Supplementary Figure 4). The ANI values between CX03 and other *L. enzymogenes* strains CX06, M497-1, YC36, C3, and OH11 were approximately 88%, and the DDH values were approximately 35%. Lower ANI and DDH values were obtained when other *Lysobacter* species were regarded as references. Only the ANI and DDH values between *L. enzymogenes* CX06 and *L. enzymogenes* strains YC36, C3 exceeded 96% and 70%, respectively.

**FIGURE 4:**
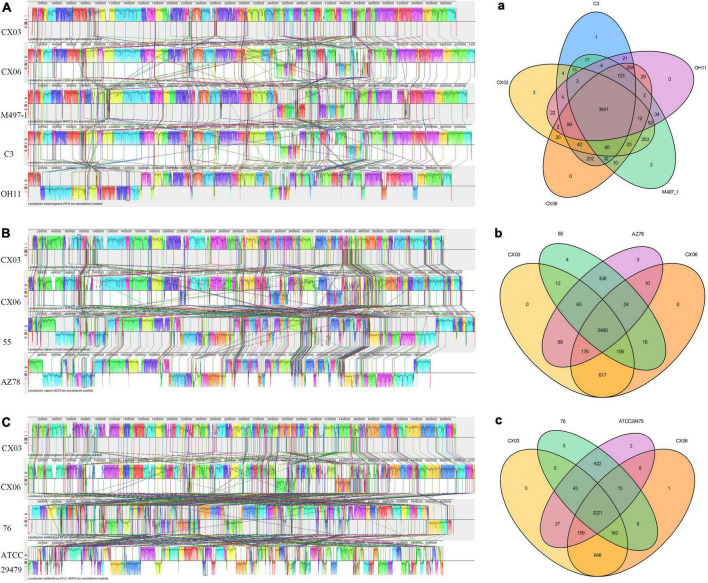
Global alignment of the genome sequences of completely sequenced *L. enzymogenes* CX03 and CX06 against other representative *Lysobacter* genome sequences. **(A)** Mauve progressive alignment of the genomes of CX03, CX06 and other representative *L. enzymogenes* strains M497-1, C3 and OH11. **(B)** Mauve progressive alignment of the genomes of CX03, CX06 and representative *L. capsici* strains 55 and AZ78. **(C)** Mauve progressive alignment of the genomes of CX03, CX06 and representative *L. antibioticus* 76, ATCC29479. Boxes with the same color indicate syntenic regions. Boxes below the horizontal strain line indicate inverted regions. Rearrangements are indicated with the colored lines. The scale is in nucleotides. **(a)** Venn diagram showing the numbers of shared and unique Clusters of Orthologous Genes among the genomes of CX03, CX06 and other representative *L. enzymogenes* strains M497-1, C3 and OH11. **(b)** Venn diagram showing the numbers of shared and unique Clusters of Orthologous Genes among the genomes of CX03, CX06 and the representative *L. capsici* strains 55 and AZ78. **(c)** Venn diagram showing the numbers of shared and unique Clusters of Orthologous Genes among the genomes of CX03, CX06 and the representative *L. antibioticus* strains 76, ATCC29479.

The whole genome sequences including *L. enzymogenes* strains CX03, CX06, M497-1, C3, OH11, *L. capsici* strains 55 and AZ78, *L. antibioticus* strains 76 and ATCC29479 were compared using Mauve. The alignments demonstrated that obvious local collinear block (LCB) inversion was detected among the five *L. enzymogenes* strains CX03, CX06, M497-1, C3, and OH11, but the CX06 genome was highly syntenic with M497-1. At the species level, more gene insertion or deletion occurred between *L. enzymogenes* strains CX03, CX06 and *L. capsici* strains 55, AZ78 or *L. antibioticus* strains 76, ATCC29479, which indicated the far genetic distances of the different *Lysobacter* species (Figures 4A–C).

A Venn diagram indicating the specific genes among *L. enzymogenes* strains CX03, CX06, M497-1, C3, OH11, *L. capsici* strains 55, AZ78 and *L. antibioticus* strains 76, ATCC29479 was constructed (Figure 4). In total, 3,641 homologous genes existed in *L. enzymogenes* strains CX03, CX06, M497-1, C3, and OH11. CX03 shared 4,092 orthologous genes with M497-1, 3,934 orthologous genes with CX06, 3,901 orthologous genes with C3, and only 3,835 orthologous genes with OH11. Among the five *L. enzymogenes* strains, three unique genes were found in the CX03 genome, two unique genes were found in the M497-1 genome, one unique gene was found in the C3 genome, and no unique genes was observed in the genomes of strains CX06 and OH11. Moreover, 3,460 orthologous genes were found among the genomes of strains CX03, CX06, and *L. capsici* strains 55, AZ78. CX03 shared 3,693 orthologous genes with 55, and shared 3,770 orthologous genes with AZ78. Intriguingly, four unique genes were found in strain 55, three unique genes were found in strain AZ78, and no unique genes were found in strains CX03 and CX06. Furthermore, 3,221 orthologous genes were found among the genomes of strains CX03, CX06 and *L. antibioticus* strains 76, ATCC29479. CX03 shared 3,431 orthologous genes with 76, and shared 3,450 orthologous genes with ATCC29479. In addition, five unique genes were found in the 76 genome, two unique genes were found in the ATCC29479 genome, one unique gene was found in the CX06 genome, but no unique genes were found in the CX03 genome.

### Functional genes related secondary metabolites and microbe–plant interactions

#### Gene clusters involved in the biosynthesis of antibiotics

**FIGURE 5:**
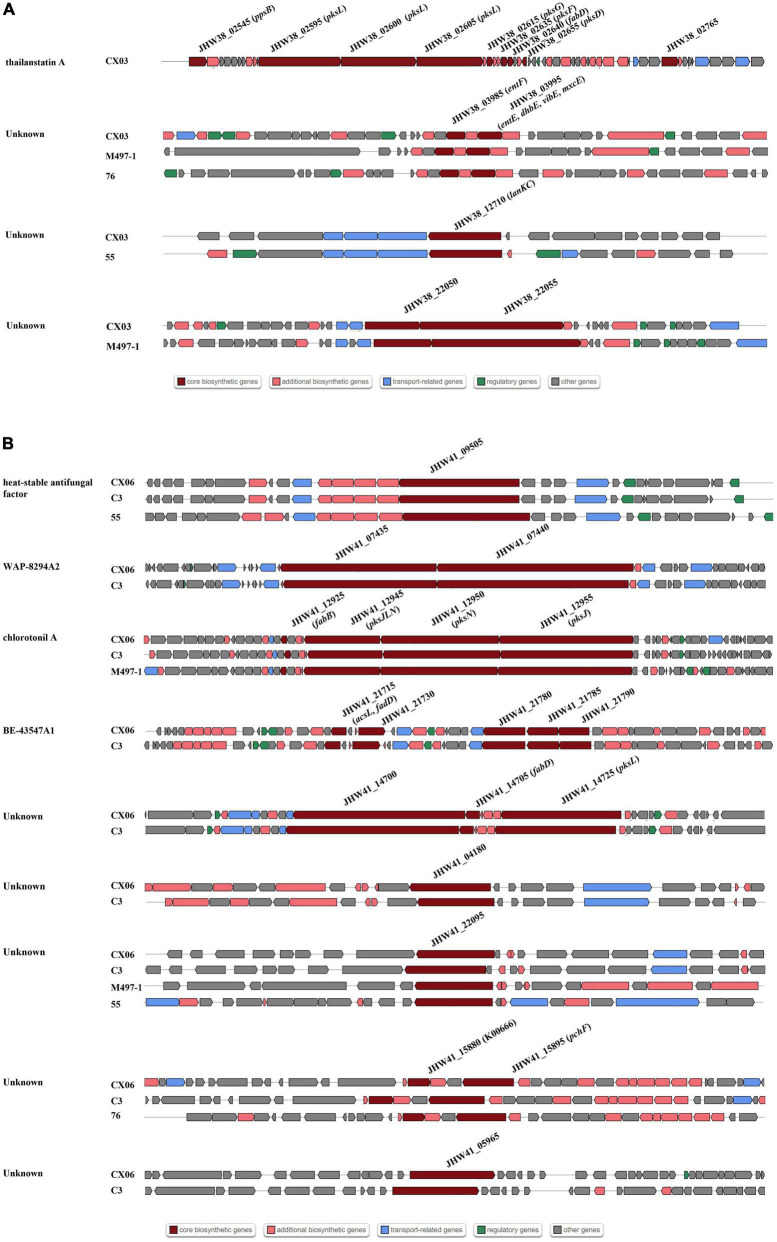
Comparisons of gene clusters for secondary metabolites synthesis with other representative *Lysobacter* strains C3, M497-1, 55 and 76. **(A)** Gene clusters existed in strain CX03, not in strain CX06. **(B)** Gene clusters existed in strain CX06, not in strain CX03. Dark red indicated the core biosynthetic genes in different gene clusters. The core biosynthesis genes were marked in the diverse gene clusters.

To compare the gene clusters involved in antibiotic synthesis in *L. enzymogenes* CX03 and CX06, four *Lysobacter* strains including *L. enzymogenes* M497-1, *L. enzymogenes* C3, *L. capsici* 55, and *L. antibioticus* 76 were selected. For strain CX03, 11 gene clusters associated with secondary metabolite biosynthesis were detected, including six gene clusters encoding NRPSs (non-ribosomal peptide synthetases), two gene clusters encoding lanthipeptide, one gene cluster encoding arylpolyene, one gene cluster encoding RiPP-like and one redox-cofactor (Table 1). Moreover, 16 gene clusters linked with secondary metabolite biosynthesis were searched in the CX06 genome, including ten gene clusters encoding NRPSs, three gene clusters encoding lanthipeptide, one encoding RiPP-like protein, one arylpolyene and one redox-cofactor. According to the anti-SMASH database, seven secondary metabolites including xanthomonadin 1, Le-pyrrolopyrazines, lankacidin C, and four unknown antibiotics existed in strains CX03, CX06 and C3 with high homology (Supplementary Figure 5) (Table 1). Four secondary metabolites including thailanstatin A and three unknown antibiotics were detected in strain CX03, but not in strain CX06 (Figure 5A). Nine secondary metabolites including HSAF, WAP-8294A2, chlorotonil A, BE-43547A1, and three unknown antibiotics existed in strain CX06, but not in strain CX03 (Figure 5B). Interestingly, the gene cluster encoding thailanstatin A was detected only in the CX03 genome, not in the genomes of strains CX06, C3, M497-1, 55, 76 (Table 1). An unknown secondary metabolite with core coding genes *entF* and *entE* was found in the genomes of *L. enzymogenes* strains CX03, M497-1 and *L. antibioticus* 76, but not in *L. enzymogenes* strains CX06, C3 and *L. capsici* 55. Another unknown secondary metabolite with the core coding gene *lanKC* was only detected in *L. enzymogenes* CX03 and *L. capsica* 55, but not in other *Lysobacter* strains (Figure 5A). In addition, many gene clusters associated with the biosynthesis of different secondary metabolites were only found in *L. enzymogenes* strains, not in *L. antibioticus* or *L. capsici* strains, such as Le-pyrrolopyrazines, thailanstatin A, WAP-8294A2, chlorotonil A, BE-43547A1 and four unknown antibiotics (Figure 5 and Supplementary Figure 5). Notably, two secondary metabolites (HSAF and WAP-8294A2) that have been widely studied were not detected in the genomes of *L. enzymogenes* CX03 and *L. antibioticus* 76 (Figure 5).

**TABLE 1:** Comparison of core gene clusters involved in secondary metabolites biosynthesis in strains CX03, CX06, MA97-1, C3, 55 and 76.

Antibiotic name	Type	Core gene clusters	Size	Position	CX03	CX06	C3	M497-1	55	76
Xanthomonadin I	Arylpolyene	*fabB*	41,200	386,889–428,088	JHW38_18245–JHW38_18410	JHW41_01600–JHW41_01760	GLE_RS21925–GLE_RS22085	LEN_RS02150–LEN_RS02315	LC55x_RS22660-LC55x_RS22790	NA
Le-pyrrolopyrazines	NRPS; RiPP-like	K09930	58,001	2,518,673–2,576,673	JHW38_05710–JHW38_05915	JHW41_10560–JHW41_10760	GLE_RS12970–GLE_RS13160	LEN_RS10150–LEN_RS10345	NA	NA
Lankacidin C	Redox-cofactor	*pqqCDE*	22,191	1,625,895–1,648,201	JHW38_09520–JHW38_09630	JHW41_07015–JHW41_07120	GLE_RS16545–GLE_RS16650	NA	LC55x_3908–LC55x_3932	LA76x_RS17710–LA76x_RS17810
Heat-stable antifungal factor	T1PKS; NRPS	NA	49,486	2,263,501–2,312,986	NA	JHW41_09425–JHW41_09585	GLE_RS14120–GLE_RS14275	NA	LC55x_1990–LC55x_2023	NA
WAP-8294A2	NRPS	*mbtH, nocI*	89,028	1,728,431–1,817,458	NA	JHW41_07340–JHW41_07515	GLE_RS16150–GLE_RS16310	NA	NA	NA
Chlorotonil A	NRPS	*pksJLN, fabB*	47,083	3,892,265–3,939,347	NA	JHW41_12850–JHW41_13055	GLE_RS10810–GLE_RS11030	LEN_RS12320–LEN_RS12520	NA	NA
BE-43547A1	NRPS, lanthipeptide-class-ii	*gtrB, csbB, fadD*	67,920	5,253,420–5,321,339	NA	JHW41_21620–JHW41_21865	GLE_RS02295–GLE_RS02540	NA	NA	NA
Thailanstatin A	NRPS, PKS-like, T3PKS, transAT-PKS	*ppsB, pksDFGL, fabD*	123,854	540,483–664,336	JHW38_02545–JHW38_02810	NA	NA	NA	NA	NA
Unknown	RiPP-like	K09930	9,371	2,167,353–2,176,723	JHW38_07405–JHW38_07465	JHW41_09025–JHW41_09080	GLE_RS14610–GLE_RS14670	LEN_RS08690–LEN_RS08745	LC55x_1902–LC55x_1914	LA76x_RS08985–LA76x_RS09040
Unknown	NRPS	*entF*	41,663	4,398,433–4,440,095	JHW38_18540–JHW38_18725	JHW41_01905–JHW41_02080	GLE_RS21600–GLE_RS21775	LEN_RS22320–LEN_RS22485	NA	LA76x_RS01375–LA76x_RS01565
Unknown	NRPS	*tssF*	81,718	1,060,275–1,141,992	JHW38_04265–JHW38_04500	JHW41_12565–JHW41_12740	GLE_RS11135–GLE_RS11310	NA	NA	LA76x_RS12285–LA76x_RS12440
Unknown	Lanthipeptide-class-ii	*ydhP*	22,877	4,548,972–4,571,848	JHW38_19190–JHW38_19295	JHW38_19190–JHW38_19295	GLE_RS10630–GLE_RS10710	NA	NA	LA76x_RS12285–LA76x_RS12440
Unknown	NRPS	*entF, entE, dhbE, vibe, mxcE*	41,926	966,828–1,008,753	JHW38_03880–JHW38_04055	NA	NA	LEN_RS11870–LEN_RS11980	NA	LA76x_RS11825–LA76x_RS12000
Unknown	Lanthipeptide-class-iii	*lanKC*	22,730	3,031,820–3,054,549	JHW38_12680–JHW38_12755	NA	NA	NA	LC55x_RS10165–LC55x_RS10235	NA
Unknown	NRPS, T1PKS	*fadEG, ampC*	59,610	5,194,324–5,253,933	JHW38_21965–JHW38_22130	NA	NA	LEN_RS18810–LEN_RS18960	NA	NA
Unknown	NRPS, transAT-PKS	*fabD, pksL*	82,610	3,582,829–3,658,580	NA	JHW41_14645–JHW41_14785	GLE_RS09115–GLE_RS25730	NA	NA	NA
Unknown	Lanthipeptide-class-ii	*yojI*	21,693	973,830–995,522	NA	JHW41_04130–JHW41_04225	GLE_RS19595–GLE_RS19685	NA	NA	NA
Unknown	Lanthipeptide-class-ii	*fimA*	22,877	5,359,830–5,382,706	NA	JHW41_22055–JHW41_22145	GLE_RS02010–GLE_RS02095	LEN_RS21820–LEN_RS21890	LC55x_RS21600–LC55x_RS21695	NA
Unknown	NRPS	K0066, *pchF*	47,083	3,892,265–3,939,347	NA	JHW41_15810–JHW41_15980	GLE_RS08020–GLE_RS08165	NA	NA	LA76x_RS14890–LA76x_RS15050
Unknown	NRPS	*queCE*	43,547	1,365,062–1,408,608	NA	JHW41_05845–JHW41_06035	GLE_RS17615–GLE_RS17765	NA	NA	NA

NA, not available.

#### Bacterial secretion system

Comparative genomic analysis of the bacterial secretion systems among strains CX03, CX06, M497-1, C3, 55 and 76 showed that *L. enzymogenes* CX03 harbored a highly conserved T2SS (type II secretion system) gene cluster (*gspDEFGHIJKLM*) (Supplementary Figure 6), covering 11,589 bp with 10 ORFs. All the core genes were found in *Lysobacter* strains CX06, M497-1, C3, 55 and 76, and shared a high consistency at the amino acid level (exceeding 80%) (Supplementary Table 5).

A large *sct*/*hrp* gene cluster of ten genes (*sctJLQSTUV*, *yscN*, *hrpAB*) was searched in the genome of *L. enzymogenes* CX03, and shared high similarities with other *Lysobacter* strains (exceeding 80%), except the gene *sctQ* encoding cytoplasmic ring protein SctQ of the type III secretion system, which showed a low sequence identity with strains 55 and 76 (74% and 64%, respectively) (Supplementary Table 5 and Supplementary Figure 6).

Moreover, ten key genes related to T4SS (*virB1,2,3,4,6,8,9,10,11* and *virD4*) were all found in strains CX03, CX06, M497-1, C3, 55 and 76 (Supplementary Figure 6). However, only four genes (*virB4*, *virB9*, *virB11*, and *virD4*) retrieved from the CX03 genome shared a high similarity (identity > 80%) with other four *Lysobacter* strains, while other genes shared a low sequence identity (below <80%). In particular, the sequence identity of the *virB6* gene was less than 70% between CX03 and M497-1, C3, 55, 76, and was only 37% between CX03 and CX06 (Supplementary Table 5).

T6SS widely existed in many Gram-negative bacteria. In this study, 28 genes linked with the T6SS were only found in the genomes of strains CX06 and C3, not in CX03, M407-1, 55 and 76 (Supplementary Figure 6). The 28 genes were detected to encode different putative proteins, including 12 core genes (*tssABCHEFGMLK*, *tagFH*) for type VI secretion system proteins, and two genes (*vgrG* and *hcp*) related to extracellular structural components of the secretion machine and specific effectors. Furthermore, most T6SS genes were multicopy in strains CX06 and C3 (Supplementary Table 5).

In addition, 11 genes associated with the Sec system were detected in the CX03 genome, and possessed high sequence identities with those of the other five *Lysobacter* strains, except for the *secG* gene, which only showed 66% and 63% identities between CX03 and strains 55, 76. Three genes (*tolC*, *hlyB* and *hlyD*) associated with the T1SS were found in the genome of CX03, but the genes *hylB* and *hlyD* were absent in the genomes of CX06, C3 and 55. Three genes (*tatABC*) involved in twin arginine targeting were all present in the genomes of CX03, CX06, M497-1, C3, 55 and 76; however the identities of the corresponding genes between CX03 and other *L. enzymogenes* strains were higher than those between CX03 and *L. capsici* 55, *L. antibioticus* 76 strains (Supplementary Table 5 and Supplementary Figure 6).

#### Flagella, type IV pilus and chemotaxis

The comparative genomic analysis showed that sixteen key genes (mainly the *fli* and *flg* gene clusters) associated with flagella biosynthesis were searched in the *L. enzymogenes* CX03 genome. Most genes encoding flagella-associated proteins were also detected in *L. enzymogenes* CX06, M497-1, C3 and *L. capsici* 55, while only three genes (*fliH*, *fliI* and *fliO*) were detected in the genome of *L. antibioticus* 76, and the gene *flgD* was not detected in the *L. enzymogenes* CX06 genome. The identities of these genes between CX03 and CX06, M497-1, and C3 were higher than those between CX03 and 55, 76. Most genes detected in the CX03 genome shared higher amino acid similarities with *L. enzymogenes* CX06 and C3, but showed a low similarity with that in M497-1. Only one gene *fliI* shared a high sequence identity (exceeding 90%) among the *Lysobacter* strains CX03, CX06, M497-1, C3, 55 and 76 (Supplementary Table 6). In addition, 15 genes (*pilWECVGABDUMOPQT* and *fimT*) involved in type IV pilus were found in the genomes of strains CX03, CX06, MA97-1, C3, 55 and 76 (Supplementary Table 6). Most of these genes from strain CX03 shared high homology with those in other *Lysobacter* stains at the amino acid level (identities > 80%). However, the identities of *pilE*, *pilC*, *pilV*, *pilA* and *fimT* genes between CX03 and other *Lysobacter* strains were less than 70%. Similarly, *pilW* in strain CX03 shared higher identities with *L. enzymogenes* strains CX06, M497-1 and C3, but showed a low similarity with *L. capsici* 55 and *L. antibioticus* 76. Moreover, 7 genes (*cheW*, *pilJ*, *pilI*, *cheB*, *wspD*, *wspB*, *wspA*) involved in chemotaxis were sought in the genome of *L. enzymogenes* CX03, and shared a high homology with the other three *L. enzymogenes* strains (>80%) at the amino acid level. However, the *wspD* gene encoding purine-binding chemotaxis protein CheW retrieved from the genome of strain CX03 did not exist in strain CX06, and exhibited very low identities with those in strains 55 and 76 (Supplementary Table 6).

#### Two-component system

In addition, 25 TCSs (two-component system) were searched in the genome of strain CX03, most of which were highly similar to those of strains CX06, M497-1, C3, 55 and 76. Based on the topological characteristics of sensor histidine kinase (HK) and response regulator (RR), the 31 TCSs were assigned to diverse subfamilies. For, example, phosphate regulation TCSs such as phoR/B and phoQ/P were detected in the genomes of strains CX03, CX06, M497-1, C3, 55 and 76, with amino acid sequence identities exceeding 93% (Supplementary Table 7). The TCSs *kdpD/E*, *algR/Z*, and *pilH/G* retrieved from CX03 shared high similarity with those in the other five *Lysobacter* strains (>90 identity). Nevertheless, the identities of *wspA/B*, *wspD/E* and *wspF/R* which were associated with chemotaxis between CX03 and CX06, M497-1, and C3 (>80%), were higher than those between CX03 and 55, 76 (<70%) (Supplementary Table 7). Interestingly, the *rpf C/F* TCS in strain CX03 owned a high similarity with those in strains CX06 and C3 (92% and 91%, respectively), but exhibited a low identity to strain M497-1 (47% and 48%, respectively). Furthermore, the *mdt* TCS was only found in the genomes of *L. enzymogenes* strains CX03 and M497-1 with the sequence identities exceeding 80% and most were multicopy genes. Although two genes *mdtAC* were also found in the genome of *L. capsici* 55, their identities with those in *L. enzymogenes* CX03 at the amino acid level were very low (41% and 77%, respectively). The *rcs* TCSs were not detected in strains *L. enzymogenes* CX06 and *L. antibioticus* 76, and low homology of genes *rcsC* and *rcsB* was found between CX03 and C3, M497-1, 55 strains, especially the sequence identity of *rcsC* gene was less than 80% (Supplementary Table 7).

#### Quorum sensing

The comparative genomic analysis showed that 40 genes associated with quorum sensing (QS) were retrieved in the CX03 genome. In this study, the key gene *expR*, which was related to *N*-acyl homoserine lactones (AHLs), was observed in strain CX03 and shared a high homology with other *Lysobacter* strains. In addition, the QS gene clusters, *tox*, *rpf*, *phn* and the gene *hfq* were highly conserved among strains CX03, CX06, and the other four *Lysobacter* strains (Supplementary Table 8). However, many genes shared a higher sequence identity between CX03 and CX06, M497-1, C3 than that between CX03 and 55, 76 (Supplementary Table 8).

#### Selenium metabolism

In this study, *L. enzymogenes* CX03 and CX06 were tested to generate elemental Se nanoparticles (SeNPs) from Na_2_SeO_3_. The highest concentration of Na_2_SeO_3_ tolerated for strains CX03 and CX06 was 75 mM, and the production of SeNPs achieved a maximum when the concentration of Na_2_SeO_3_ was 5 mM (Supplementary Figure 7). Whole genome annotation demonstrated that many genes linked with selenium metabolism were found in the genomes of *L. enzymogenes* CX03 and CX06, and shared high similarity with *Lysobacter* strains M497-1, C3, 55 and 76. Among these genes, eight genes (*metB*, *metC*, *metE*, *metG*, *metH*, *mdeA*, and *trxB*) were related to selenium association, and two genes (*cysN* and *cysD*) were involved in selenium transportation. However, the identities of these genes between CX03 and *L. enzymogenes* strains CX06, M497-1, C3 (90–98%) were higher than those between CX03 and *L. capsici* 55, *L. antibioticus* 76 (83–96%) (Supplementary Table 9).

#### Biosynthesis of siderophores

In addition, the result showed that eight key genes (*entACDFE*, *dhbA*, *mcyF*, and *racD*) associated with the biosynthesis of siderophore group non-ribosomal peptides were found in the genomes of strains CX03, CX06, M497-1, C3, 55 and 76. Most genes in strain CX03 shared high sequence identities (exceeding 70%) between strains CX06, M497-1, C3 at the amino acid level, except the gene *entD* encoding 4′-phosphopantetheinyl, which showed low homology between CX03 and CX06, M497-1, C3, 55 and 76 (<50% identity) (Supplementary Table 10).

#### Polyketone, lipopolysaccharide, peptidoglycan biosynthesis

Moreover, 42 putative protein coding genes involved in the biosynthesis of polyketone, lipopolysaccharide and peptidoglycan were searched in the genome of *L. enzymogenes* CX03 and CX06, including four genes (*rfbABCD*) related to polyketide sugar unit biosynthesis, 17 genes (mainly the *lpx* gene cluster) related to lipopolysaccharide biosynthesis, 19 genes (mainly the *mur* gene cluster) related to peptidoglycan biosynthesis, and two genes *proA*, *proB* related to carbapenem biosynthesis. All these genes possessed high homology with those of the other five *Lysobacter* strains at the amino acid level, except the gene *rfbD*, which showed low homology between CX03 and 55, 76 (69% and 71% identities, respectively) (Supplementary Table 11).

## Discussion

*Lysobacter enzymogenes* has been widely reported to have antagonistic abilities against diverse plant-pathogenic fungi due to its ability to produce various antifungal compounds and multiple lytic enzymes. In this study, two *L. enzymogenes* strains exhibited distinct antipathogenic activities; strain CX03 showed broad-spectrum antagonistic capacities against various plant-pathogenic bacteria, while strain CX06 showed broad-spectrum inhibiting abilities toward multiple plant-pathogenic fungi. To explore the possible biocontrol mechanisms for the different antipathogenic capacities of the two *L. enzymogenes* strains, especially the potential biocontrol mechanism of strain CX03 inhibiting bacteria, the complete genomes of the two strains were sequenced and comparative genomic analysis among different *Lysobacter* strains was performed.

Based on the phylogenetic analysis, *L. enzymogenes* strains clustered into two subgroups indicating the high diversity of the species, which was consistent with a previous study (Gómez Expósito et al., 2015). ANI and DDH were widely used to calculate the similarity of bacteria, however, the ANI and DDH values between strain CX03 and other *L. enzymogenes* strains CX06, M497-1, YC36, C3, and OH11 were below 95% and 70%, respectively. Only the ANI and DDH values between CX06 and *L. enzymogenes* strains YC36, C3 were greater than 95% and 70%, respectively. Even though the ANI and DDH values between strains M497-1 and YC36, C3, OH11 which had been classified as *L. enzymogenes*, were still below 95% and 70%, respectively. These results may indicate that ANI and DDH was not suitable for the classification in *Lysobacter* strains. Pairwise alignment of whole genomes played an important role in the diversity analysis of lineages in different species, especially for the evolution of species and subspecies (Nowell et al., 2014). Thus, the obvious gene transfer or large local collinear block inversion between CX03, CX06 and 55, AZ78, 76, ATCC29479 indicated that these strains belonged to different *Lysobacter* spp., supporting the phylogenetic result that these strains were in different clades. In addition, more orthologous genes were detected between CX03, CX06 and *L. enzymogenes* strains than between CX03, CX06 and *L. capsici* or *L. antibioticus* strains, which proved the above result of the phylogenetic analysis that strains CX03 and CX06 were classified as *L. enzymogenes*, followed by *L. antibioticus* strains, *L. capsici* strains successively.

*Lysobacter* was a bacterial genus known to generate a number of antibiotics (Hashizume et al., 2016). However, the antimicrobial activities of different *Lysobacter* spp. were significantly different. Previous studies showed that most *L. enzymogenes*, *L. antibiotics*, *L. capsica* and *L. gummosus* strains had strong antagonistic abilities toward many fungi and oomycetes, but only a few *L. antibioticus* strains exhibited antagonistic activity against the bacteria *X. campestris* pv. *campestris* (Gómez Expósito et al., 2015). However, in our study, *L. enzymogenes* CX03 showed broad-spectrum antagonistic capacities toward plant-pathogenic bacteria and an obvious influence in controlling black rot on cabbage. In fact, most reported secondary metabolites synthesized by *L. enzymogenes* were reported to show antagonistic activities against plant fungal pathogen. *L. enzymogenes* C3 was demonstrated to suppress diseases caused by multiple fungal pathogens, including *B. sorokiniana* (Zhang and Yuen, 1999), *F. graminearum* (Jin et al., 2003), *R. solani* (Giesler and Yuen, 1998), *U. appendiculatus* (Yuen et al., 2001), *P. ultimum* (Kobayashi et al., 2005), and *Magnaporthe poae* (Kobayashi and Yuen, 2005). The main antifungal factor in strain C3 was HSAF, which had a novel structural features and inhibited against many fungal species (Yu et al., 2007). HSAF could disrupt the polarized growth of fungi by targeting the biosynthesis of sphingolipids, which were essential components of eukaryotic cell membranes and signaling molecules associated with numerous cellular process (Li et al., 2006). Genes related to hybrid polyketide synthase-non-ribosomal peptide synthetase (PKS-NRPS) were reported as key genes for the production of HSAF. The destruction of the PKS-NRPS gene in strain C3 lost the capacity to produce HSAF and reduce the ability to restrain fungal growth (Yu et al., 2007; Li et al., 2012). HSAF production was also proved to be a crucial mechanism to control cyst nematodes in *L. enzymogenes* C3 (Yuen et al., 2018). The comparative genomic analysis showed that the genes and gene clusters involved in secondary metabolite synthesis were significantly different among the six *Lysobacter* strains. As expected, a gene cluster for the biosynthesis of HSAF was found in the CX06 and C3 genomes, but not in CX03 genome, corresponding to the antifungal activity of *L. enzymogenes* CX06. The WAP-8294A compound was reported to antagonize Gram-positive organisms, and the NRPSs comprised 45 functional domains, which were related to the assembly of the 12 modules of the WAP-8294A antibiotic (Chen et al., 2015). *L. enzymogenes* OH11 was reported to produce WAP-8294A2, a cyclic lipodepsipeptide with potent anti-*S. aureus* ability (Zhang et al., 2011). However, very few studies reported its antagonistic activity against plant pathogenic bacteria. The WAP biosynthetic gene cluster in the genome of *L. enzymogenes* OH11 was identified with two large NRPS genes, WAPS1 and WAPS2 (Chen et al., 2015), and further knockdown of ORF3, ORF4, and ORF8 genes reduced the antagonistic ability of the mutant strain against Gram-positive bacteria (Zhang et al., 2011). *L. enzymogenes* CX03 showed antagonistic activities against *C. michiganensis*, but no genes or gene clusters responsible for the biosynthesis of WAP-8294A2 were found in the genome of strain CX03. Moreover, although genes or gene clusters associated with WAP-8294A2 were found in the CX06 genome, strain CX06 did not show antagonistic activity against plant pathogenic bacteria. These results might indicate that the inhibitory on *C. michiganensis* of *L. enzymogenes* CX03 was not related to WAP-8294A2. The antiSMASH analysis showed that four secondary metabolites (thailanstatin A and three unknown compounds) were found in strain CX03, not in strain CX06, especially thailanstatin A, which was only detected in strain CX03 and not in other *Lysobacter* strains. Thailanstatin A (TST-A) was first reported as an effective antiproliferative natural product generated from *Burkholderia thailandensis* MSMB43 (Liu et al., 2016). Thailanstatins were biosynthesized by a hybrid polyketide synthase-non-ribosomal peptide synthetase pathway, and the biosynthetic gene cluster was similar to that of FR901464, a prototype spliceosome inhibitor produced by *Pseudomonas* sp. 2663. It was also proven to inhibit pre-mRNA splicing (a new mechanism of anti-neoplastic action) as potently as FR901464 (Liu et al., 2013). Moreover, several *pks* genes involved in the biosynthesis of bacillaene polyketides were found in the genome of strain CX03. Polyketides were reported as a large family of secondary metabolites with antibacterial, immunosuppressive and antitumor activities (Chen et al., 2006). Interestingly, only strain CX03 exhibited direct antagonistic activities against many bacteria, while the other *L. enzymogenes* strains CX06, C3 and M497-1 did not exhibit antibacterial activities. Thus, thailanstatin A may be an unusual antibiotic that showed antibacterial activities, though the specific biological function of thailanstatin A remained to be confirmed. In addition, a core gene *lanKC* related to unknown secondary metabolite encoding class III lanthionine synthetase was found in the genome of strain CX03. Previous study showed that lanthionine synthetase complex was involved in the modification and transport of lantibiotics which displayed potent antimicrobial activity (Lu et al., 2012). Notably, phenazine antibiotics produced by *L. antibioticus* were reported to have antagonistic activities against several bacteria (Puopolo et al., 2014), however, no definite genes or gene clusters associated with the biosynthesis of phenazine were retrieved from the genome of *L. enzymogenes* CX03.

In addition, five other secondary metabolites (Chlorotonil A, BE-43547A1 and five unknown antibiotics) were found in strain CX06, but not in strain CX03. Chlorotonil A, a tricyclic macrolide generated by *S. cellulosum*, was first described in 2008 (Gerth et al., 2008), and was tested to exhibit possible antimalarial activity (Held et al., 2014). BE-43547A1 was isolated as a member of seven macrocyclic dipeptides families from *Streptomyces* strain A43547 in 1998 with different congeners A1, A2, B1, B2, C1, and C2. The BE-4357 members were found to exhibit obvious hypoxia-selective growth-inhibitory ability toward pancreatic cancer cells (Villadsen et al., 2017), and had structural similarities to the cytotoxic natural products rakicidins A and B (McBrien et al., 1995). Given the potential fact that HSAF was the main antibiotic for antagonizing plant pathogenic fungi, the specific functions of these five secondary metabolites need to be further verified.

Furthermore, seven secondary metabolites (xanthomonadin I, Le-pyrrolopyrazines, Lankacidin C and four unknown compounds) were found in strains CX03 and CX06. Xanthomonadin was first reported in *Xanthomonas*, and was unimportant during pathogenesis (Rajagopal et al., 1997). To date, the function of xanthomonadin was unknown, but it was proven to provide protection against photodynamic lipid peroxidation in liposomes and could be used for protection against photodamage (Poplawsky et al., 2000). Lankacidin C, also named bundling A or T-2636 C, was described as an antitumor antibiotic that was isolated independently from diverse *Streptomyces* species. Lankacidins have been reported to have powerful antimicrobial abilities against many Gram-positive bacteria, including some strains resistant to conventional macrolide antibiotics (Kende et al., 1995). Given that CX03 and CX06 showed distinct antagonistic activities toward plant pathogens, the seven secondary metabolites played dispensable roles in antimicrobial activity. Worth mentioning, many gene clusters related to unknown secondary metabolites were identified in the *L. enzymogenes* genomes, and these secondary metabolites could be a potential resource for novel antibiotics; however, in-depth chemical and functional analyses are needed to verify this possibility.

Siderophores, which were mostly encoded by NRPS gene clusters, have been shown to play a crucial role in iron competition with other microorganisms, and could be synthesized by *Lysobacter* strains. For example, *L. enzymogenes* C3 exhibited iron-chelating ability on CAS plate, and the iron availability was related to the antibacterial capacity (de Bruijn et al., 2015). The potential biocontrol agent *L. enzymogenes* LE16, which showed broad antagonistic activities against many pathogenic bacteria, could produce siderophores, proteases, and phosphatases in pure culture (Chen et al., 2020). *L. capsica* AZ78, which has considerable potential for the biocontrol of phytopathogenic microorganisms, harbored specific genes involved in the production of siderophores (Gerardo et al., 2016). *L. antibioticus* HS124, which showed antifungal activity against *P. capsici* was reported to produce catechol type siderophores, and the antifungal ability when Fe (III) was added was approximately 1.5 times higher than that in the absence of Fe (III) (Ko et al., 2011). Many siderophores have been widely described, such as enterobactin, a triscatechol derivative of a cyclic triserine lactone, which was achieved by non-ribosomal peptide synthetases (Raymond et al., 2003). Previous studies showed that six Ent proteins, EntA-F were related to the synthesis of enterobactin. For instance, EntF, an N-terminal elongation/condensation domain was reported as a catalyst in enterobactin assembly. EntD was known as a posttranslational modification catalyst and the EntD-mediated conversion of apo-forms of EntB and EntF to phosphopantetheinylated played important roles in the acyl activation and transfer of enterobactin assembly (Gehring et al., 1998). Pyochelin was described as a type of thiazolines siderophore which was also assembled by NRPS. Among the two gene clusters *pchEF* and *pchDCBA* which related to the pyochelin assembly, *pchF* was required for production of the entire pyochelin molecule (Quadri et al., 1999). In this study, CX03 and CX06 could produce siderophores on CAS agar plates, and *ent* gene cluster related to the production of enterobactin were found in the CX03 and CX06 genomes. So, enterobactin might be one of the siderophores produced by strains CX03 and CX06. In addition, the *pchF* gene was also found in strain CX06, considering its important role in the siderophore assembly, pyochelin might be another siderophore produced by strain CX06. However, these guesses need further experiments to verify.

*Lysobacter* members were well-known for their ability to produce a variety of extracellular enzymes, such as chitinases, elastases, glucanases, endonucleases, proteases and lipases (Zhang et al., 2001; Gökçen et al., 2014; Gómez Expósito et al., 2015). Chitinolytic activity has been proven to be one of the mechanisms related to biological control in *L. enzymogenes*. For example, the chitinolytic in *L. enzymogenes* C3 could cause conidial deformation and abnormal germ tube formation in fungi (Zhang and Yuen, 2000). *L. enzymogenes* C3 was reported to generate three extracellular β-1,3-glucanases, which were encoded by the *gluA*BC genes, and the inhibiting ability of strain C3 against *Pythium* damping-off and *Bipolaris* leaf spot decreased when the three genes were deleted (Palumbo et al., 2005). Proteolytic ability in *Lysobacter* strains has attracted most attention for its potential application in industrial processes (Gökçen et al., 2014) and was reported to inhibit phytopathogenic bacteria such as *Erwinia carotovora* (Vasilyeva et al., 2014). In this study, strains CX03 and CX06 were tested to produce cellulase and phosphatases, which might indicate their potential applications in biological control.

Previous studies have shown that various secretion systems exist in Gram-negative bacteria, and most have been identified in *Lysobacter* strains (Tseng et al., 2009). The secretion systems ranged from single transporters to multi-component complexes and were divided into six types in Gram-negative bacteria, type I to type VI secretion systems (Costa et al., 2015). T2SS was well-conserved and harbored 12–15 components calling Gsp proteins (GspA to GspO and GspS), which was crucial for bacterial survival and growth in a host or in an environment niche (Filloux, 2004; Korotkov et al., 2012). T2SS in *Lysobacter* genomes was reported to secrete cell wall degrading enzymes (Cianciotto, 2005), which may be responsible for the behavior of *Lysobacter* strains to lyse the cells of many microorganisms (Christensen and Cook, 1978). In this study, CX03 and CX06 harbored 10 *gsp* genes encoding T2SS components, which were highly conserved in *Lysobacter* strains; thus, Gsps may be linked to protein secretion and transportation, but the role of T2SS in *L. enzymogenes* CX03 and CX06 needs to be confirmed in the future.

The type III secretion system (T3SS) had been reported to translocate bacterial proteins into host cells (Galan and Wolf-Watz, 2006). T3SS was found in various pathogenic Gram-negative bacteria such as *Salmonella*, *Shigella*, *Yersinia* and *Pseudomonas*, and was responsible for protein transportation across the inner bacterial membrane (Dale et al., 2002; Ghosh, 2004). Recent studies have shown that the distribution of T3SS was not limited to pathogens, but also existed in certain endosymbiotic bacteria or biocontrol bacteria (Dale et al., 2001). For example, *L. enzymogenes* C3, a bacterium demonstrated earlier to be used in biological control of fungi, was identified to contain a T3SS, which was an essential part in bacterial-fungal interactions (Reedy, 2004).

T4SS was characterized as a multiprotein complex that may deliver DNA, effectors and protein-DNA complexes to the extracellular milieu or into the eukaryotic and prokaryotic target cells (Sgro et al., 2019). The VirB/D4 T4SS in *S. maltophilia* and *X. citri* were found to be associated with the transfer of effectors lethal to bacterial competitors, playing important roles in hosts and environmental colonization (Souza et al., 2015; Bayer-Santos et al., 2019). A recent study showed that *L. enzymogenes* OH11 employed T4SS as the main contact dependent weapon to suppress other soilborne bacteria. The T4SS-mediated killing behavior of strain OH11 was proven to be essential to inhibit the plant pathogenic bacterium *P. carotovorum* (Shen et al., 2021). In this study, the virB/D T4SS was present in the conserved genetic organization in *L. enzymogenes* strains CX03, CX06, M497-1 and C3, which indicated that the T4SS may play a vital role in the antagonistic activities of the bacterium; however, further studies need to explore the function of T4SS in *L. enzymogenes*.

T6SS was functionally defined in 2006, and existed in various Gram-negative bacteria (Pukatzki et al., 2006); it has been reported to be cell envelope spanning machine that can translocate effector proteins into eukaryotic and prokaryotic cells (Ho et al., 2014). T6SS consisted of 13 conserved core genes (mainly *tss* and *tag* gene clusters) and several accessory genes including the *imp* gene cluster, and multiple copies genes *vgrG* and *hcp*, which were hypothesized to be minimally necessary for function and the conserved genes varied in different species (Chang et al., 2014). In this study, T6SS was only detected in *L. enzymogenes* strains CX06 and C3, not in strains CX03, M497-1, 55 and 76, which was consistent with the previous report that genes encoding T6SS were only searched in *L. enzymogenes* strain C3 and *L. gummosus* strain 3.2.11 (de Bruijn et al., 2015). The variation in secretion system may reveal that T6SS corresponded to the diversity of different *Lysobacter* strains, although the specific function needs further confirmation.

Selenium was a significant element in environmental remediation and human and animal health; most beneficial microorganisms have been reported to produce extracellular selenium, such as *Bacillus* and *Rahnella* strains (Zhang et al., 2018). *R. aquatilis* ZF7 and HX2 were reported to produce elemental Se nanoparticles (SeNPs) (Yuan et al., 2020), and the potential beneficial bacterium *R. aceris* ZF458 harbored abundant genes involved in selenium metabolism (Xu et al., 2022). The *cys* and *met* gene clusters were reported to be closely related to selenium biosynthesis (Zhu et al., 2018). In this study, *L. enzymogenes* CX03 and CX06 showed strong ability to produce SeNPs from Na_2_SeO_3_; many genes involved in selenium metabolism were detected in the genomes of strains CX03 and CX06, which shared a high similarity with those in the genomes of other *Lysobacter* strains, and most homologous genes were also found in the *R. aceris* ZF458 genome (Xu et al., 2022). Considering the ability to synthesize SeNPs, *L. enzymogenes* CX03 and CX06 will have wide application prospects in nano-agriculture.

*Lysobacter* was reported as non-motile cells, with gliding motility or twitching motility replacing flagellar motility as a common form of movement in *Lysobacter* strains (Siddiqi and Im, 2016). For example, *L. capsica* strains L31 and *L. enzymogenes* strains L30 showed no motility after 4 days of incubation on soft SSM agar medium, while these strains spread from the point of inoculation, similar to gliding motility after 12 days of incubation (Gómez Expósito et al., 2015). Previous studies showed that no genes related to flagella synthesis were present in the genomes of *L. antibioticus* strains, and flagellar biosynthesis in many *Lysobacter* strains was non-functional due to the lack of genes encoding essential flagellar components (de Bruijn et al., 2015; Tomada et al., 2016). For example, *L. capsica* AZ78 did not contain genes for flagellar filaments, such as *fliC*, leading to the phenotypic description that the strain was non-motile (Park et al., 2008). In addition, only genes associated with the components of the flagellar apparatus were detected in *L. enzymogenes* C3, and the genes have been proven non-functional previously (Hayashi et al., 2001). In fact, *L. enzymogenes* strains exhibited a twitching behavior that was powered by type IV pilus (T4P) (Fulano et al., 2020a). For example, in the model strain *L. enzymogenes* OH11, many pilus structural component proteins including the major pilus subunit PilA, the motor proteins PilB and the outer membrane secretin PilQ were essential for the biogenesis of T4P and the function of twitching motility (Xia et al., 2018). Other minor pilins such as PiLVWE were also reported to play important roles in the initiation of pilus assembly, and PilD was a bi-functional enzyme which performed the N-terminal methylation of mature pilins (Nguyen et al., 2015). In addition, several flagellar type III secretion system (FT3SS) components including FlhA, FlhB, FliI, and FliR in *L. enzymogenes* OH11 were reported to acquire a distinct function to control T4P-driven twitching motility (Fulano et al., 2020b). In this study, flagellar apparatus genes and many T4P related genes were found in strains CX03 and CX06, which might reveal that twitching motility was the main form of movement in strains CX03 and CX06.

Previous studies showed that the biosynthesis of many antibiotic compounds in *Lysobacter* strains was regulated by intercellular signaling or quorum sensing mediated by the diffusible signal factor (DSF)-dependent system and the Clp (cyclic AMP receptor (CRP)-like protein) regulator (He and Zhang, 2008; Deng et al., 2011). For example, the small molecule metabolite (*Le*DSF3) in *L. enzymogenes* OH11 was proven to regulate the biosynthesis of HSAF. Furthermore, *Le*DSF3 upregulated the expression of the global regulator Clp, and the knock-out of *clp* reduced HSAF production (Han et al., 2015). WspR was reported as an important diguanylate cyclase (DGC) involved in HSAF regulation in *L. enzymogenes* OH11. Phosphorylation activated DGC activity of WspR and weakened the WspR-CdgL interaction, thus contributing to the accumulation of the cdi-GMP-bound CdgL, which in turn decreased HSAF biosynthesis operon transcription (Lin et al., 2021). In addition, *Le*DSF3-regulated HSAF production was dependent on the two-component regulatory system RpfC/RpfG, which has been served as the sensor/response regulator of DSF (Slater et al., 2000). Besides, DSF and diffusible factor (DF), were two chemically distinct autoinducers (Barber et al., 1997), and both Rpf/DSF and DF signaling systems were related to the modulation of HSAF biosynthesis in *L. enzymogenes* (Qian et al., 2013). Furthermore, Clp signaling in *L. enzymogenes* OH11 played a positive role in regulating the biosynthesis of HSAF and WAP-8294A2 (Wang et al., 2014), and the mutation of the *clp* gene in *L. enzymogenes* C3 reduced the antimicrobial activity *in vitro*, and the control efficiency to *pythium* damping-off in sugar beet (Kobayashi et al., 2005). In this study, the RpfC/RpfG TCS and *clp* gene were both found in *L. enzymogenes* strains CX03 and CX06, and shared a high homology with that in C3, indicating that the genes could be associated with the biosynthesis of antibiotics, which need to be further confirmed. AHL is the most studied autoinducer in Gram-negative bacteria controlling infections of various pathogens and *expR* encodes receptor for AHL. In the study, the gene *expR* was detected in the genomes of many *L. enzymogenes* strains, however, almost all the reported members of *Lysobacter* do not produce AHL. For example, the Le0959 of *L. enzymogenes* OH11 failed to directly degard AHL, but could bind with Pcol, and this was functioned as a new quorum quenching protein (Liao et al., 2021).

## Conclusion

This study described two *L. enzymogenes* strains CX03 and CX06, with significantly different antagonistic activities, and provided a comprehensive comparative genomic analysis for the potential biocontrol mechanisms of the two strains. Strain CX03 presented a broad-spectrum antagonistic activity toward different bacteria and showed a prominent effect in controlling black rot on cabbage, while strain CX06 exhibited a broad-spectrum inhibitory ability against diverse fungi and oomycete, and showed a remarkable effect in controlling stem rot on Chinese cabbage. Whole genomic and phylogenetic analysis demonstrated that the two strains CX03 and CX06 belonged to *L. enzymogenes*. Comparative genomic analysis showed that diverse secondary metabolites with different antagonistic activities were found in *L. enzymogenes* strains CX03 and CX06. Moreover, large numbers of genes involved in siderophore biosynthesis, bacterial secretion system, quorum sensing, selenium metabolism, two-component system were also detected in the CX03 and CX06 genomes. All these features of strains CX03 and CX06 revealed new insight into the comprehensive metabolic pathways. To our knowledge, strain CX03 is one of the few *L. enzymogenes* strains with the ability to directly inhibit bacteria, and this was the first study to systematically analyze the functional genes responsible for the biosynthesis of potential antibiotics with antibacterial activity in *L. enzymogenes* strains. Overall, all these features of *L. enzymogenes* strains CX03 and CX06 indicated that the two strains would be potential biocontrol agents for plant disease control, and the study provides detailed insights for the understanding of biocontrol mechanisms in *L. enzymogenes*.

## Data availability statement

The datasets presented in this study can be found in online repositories. The names of the repository/repositories and accession number(s) can be found below: https://www.ncbi.nlm.nih.gov/genbank/ (CP067395.1 and CP067396.1); https://www.ncbi.nlm.nih.gov/ (SRR19560894 and SRR19568259).

## Author contributions

SX, LL, and BL conceived and designed the experiments. SX and ZZ performed the experiments and analyzed the data. SX and ZZ wrote the manuscript. LL, XX, YS, TF, and AC revised the manuscript. All authors have read and approved of the final version of the manuscript.

## Footnotes

1
https://pfam.xfam.org/

2
http://www.ebi.ac.uk/uniprot/

3
https://antismash.secondarymetabolites.org

## Supplementary material

Supplementary Figure 1 Production of siderophores, extracellular enzymes and general morphological characteristics, gliding motility of *L. enzymogenes* CX03 and CX06.

Supplementary Figure 2 Growth and pH curves of *L. enzymogenes* CX03 and CX06. **(A)** Colony concentrations of CX03 and CX06 at different culture times. **(B)** pH values of CX03 and CX06 suspensions at different culture times.

Supplementary Figure 3 Phylogenetic tree highlighting the relative positions of *L. enzymogenes* CX03 and CX06 among other *Lysobacter* strains. The phylogenetic tree was constructed based on four housekeeping genes (16S rRNA, *gyrB*, *atpD*, *rpoD*) according to the aligned gene sequences using maximum likelihoods derived from MEGA 6.0 software. Bootstrap values (1,000 replicates) were shown at the branch points. The scale bar indicates 0.05 nucleotide substitutions pernucleotide position.

Supplementary Figure 4 Percentage of the average nucleotide identities (ANI) and *in silico* DNA-DNA hybridization (DDH) among the selected *Lysobacter* strains. ANI values were computed for a pairwise genome comparison using the OrthoANIu algorithm. The percentage of ANI was shown on the top right. DDH values were calculated by using the Genome-to-Genome Distance Calculator (GGDC). The percentage of DDH was shown on the bottom left.

Supplementary Figure 5 Comparisons of gene clusters for secondary metabolites (both existed in CX03 and CX06) with other representative *Lysobacter* strains C3, M497-1, 55 and 76. Dark red indicated the core biosynthetic genes in different gene clusters. The core biosynthesis genes were marked in the diverse gene clusters.

Supplementary Figure 6 Comparison of the bacterial secretion system gene clusters of *L. enzymogenes* CX03 and CX06 against four previous fully sequenced *Lysobacter* genomes. **(A)** Type I secretion system. **(B)** Type II secretion system. **(C)** Type III secretion system. **(D)** Type IV secretion system. **(E)** Sec (secretion) system. **(F)** Type VI secretion system. The same color represented genes with the same or similar biological function. Arrows denoted putative transcriptional units. The length of blocks represented the size of genes (1 cm = 1000 bp).

Supplementary Figure 7 Determination of selenium synthesis capabilities of *L. enzymogenes* CX03 and CX06. **(A)** Nanoselenium formation by strains CX03 and CX06 grown in LB broth supplemented with different concentrations of Na_2_SO_3_. Error bars represent the range of standard errors. Three biological replicates were performed for each treatment, and the experiments were independently repeated three times. **(B)** The survival of strains CX03 and CX06 in different concentrations of Na_2_SO_3_.
