# Supplementary material for: Comparative genomics provides insights into the potential biocontrol mechanism of two Lysobacter enzymogenes strains with distinct antagonistic activities
Source: Front Microbiol. 2022 Aug 11;13:966986. doi: 10.3389/fmicb.2022.966986 (PMC9410377; doi:10.3389/fmicb.2022.966986)
Supplement: Supplementary file 1 [file Table_3.DOCX]

**Supplementary Table 3** Number of genes associated with general COG functional categories

| Code | *Lysobacter enzymogenes* CX03 | | *L. enzymogenes* CX06 | | Descirption |
| --- | --- | --- | --- | --- | --- |
|  | Value | % age | Value | % age |  |
| A | 2 | 0.04 | 2 | 0.04 | RNA processing and modification |
| B | 1 | 0.02 | 1 | 0.02 | Chromatin structure and dynamics |
| C | 189 | 3.78 | 191 | 3.75 | Energy production and conversion |
| D | 46 | 0.92 | 44 | 0.86 | Cell cycle control, cell division, chromosome partitioning |
| E | 264 | 5.28 | 259 | 5.09 | Amino acid transport and metabolism |
| F | 68 | 1.36 | 69 | 1.36 | Nucleotide transport and metabolism |
| G | 187 | 3.74 | 196 | 3.85 | Carbohydrate transport and metabolism |
| H | 163 | 3.26 | 172 | 3.38 | Coenzyme transport and metabolism |
| I | 204 | 4.08 | 179 | 3.52 | Lipid transport and metabolism |
| J | 263 | 5.26 | 270 | 5.31 | Translation, ribosomal structure and biogenesis |
| K | 343 | 6.87 | 308 | 6.05 | Transcription |
| L | 123 | 2.46 | 125 | 2.46 | Replication, recombination and repair |
| M | 290 | 5.80 | 292 | 5.74 | Cell wall/membrane/envelope biogenesis |
| N | 81 | 1.62 | 81 | 1.59 | Cell motility |
| O | 227 | 4.54 | 217 | 4.26 | Posttranslational modification, protein turnover, chaperones |
| P | 208 | 4.16 | 212 | 4.17 | Inorganic ion transport and metabolism |
| Q | 160 | 3.20 | 145 | 2.85 | Secondary metabolites biosynthesis, transport and catabolism |
| R | 432 | 8.65 | 424 | 8.33 | General function prediction only |
| S | 321 | 6.43 | 320 | 6.29 | Function unknown |
| T | 239 | 4.78 | 242 | 4.76 | Signal transduction mechanisms |
| U | 83 | 1.66 | 105 | 2.06 | Intracellular trafficking, secretion, and vesicular transport |
| V | 137 | 2.74 | 129 | 2.54 | Defense mechanisms |
| W | 44 | 0.88 | 39 | 0.77 | Extracellular structures |
| X | 23 | 0.46 | 20 | 0.39 | Mobilome: prophages, transposons |
| Z | 3 | 0.06 | 2 | 0.04 | Cytoskeleton |
| - | 895 | 17.91 | 1044 | 20.52 | Not in COGs |

The total % age is based on the total number of protein coding genes in the annotated genome.
