# Supplementary material for: Comparative genomics provides insights into the potential biocontrol mechanism of two Lysobacter enzymogenes strains with distinct antagonistic activities
Source: Front Microbiol. 2022 Aug 11;13:966986. doi: 10.3389/fmicb.2022.966986 (PMC9410377; doi:10.3389/fmicb.2022.966986)
Supplement: Supplementary file 2 [file Table_4.DOCX]

**Supplementary Table 4** Genomic features of *Lysobacter enzymogenes* CX03 and other *Lysobacter* sp.

| Features | *L. enzymogenes* CX03 | *L. enzymogenes* CX06 | *L. enzymogenes* M497-1 | *L. enzymogenes* C3 | *L. capsici* 55 | *L. antibioticus 7*6 |
| --- | --- | --- | --- | --- | --- | --- |
| Size (bp) | 5,947,018 | 6,206,196 | 6,096,022 | 6,157,384 | 6,391,889 | 5,916,388 |
| G+Ccontent(%) | 69.70 | 69.91 | 69.4 | 69.9 | 66.6 | 66.8 |
| Replicons | One chromosome | One chromosome | One chromosome | One chromosome | One chromosome | One chromosome |
| Total genes | 5065 | 5158 | 4932 | 5082 | 5302 | 4892 |
| Predicted no. of CDS | 4996 | 5088 | 4866 | 5012 | 5198 | 4801 |
| Ribosomal RNA | 69 | 70 | 66 | 70 | 62 | 60 |
| Transfer RNA | 59 | 60 | 56 | 60 | 52 | 50 |
| Other RNA | 4 | 4 | 4 | 4 | 4 | 4 |
| Pseudogene | 39 | 29 | 31 | 37 | 42 | 31 |
| GenBank sequence | CP067395.1 | CP067396.1 | AP014940.1 | CP013140.1 | CP011130.1 | CP011129.1 |
