# Supplementary material for: Comparative genomics provides insights into the potential biocontrol mechanism of two Lysobacter enzymogenes strains with distinct antagonistic activities
Source: Front Microbiol. 2022 Aug 11;13:966986. doi: 10.3389/fmicb.2022.966986 (PMC9410377; doi:10.3389/fmicb.2022.966986)
Supplement: Supplementary file 3 [file Table_5.DOCX]

**Supplementary Table 5** Homology analysis of bacterial secretion system in *Lysobacter enzymogenes* CX03, CX06 and other representative *Lysobacter* strains.

| **Strain** |  | ***L. enzymogenes* CX03** | | ***L. enzymogenes* CX06** | | ***L. enzymogenes* M497-1** | | ***L. enzymogenes* C3** | | ***L. capsici* 55** | | ***L. antibioticus* 76** | |
| --- | --- | --- | --- | --- | --- | --- | --- | --- | --- | --- | --- | --- | --- |
| **Genes** | **Product Definition** | **Locus Tag** | **Protein ID** | **Protein ID** | **Homology (%)** | **Protein ID** | **Homology (%)** | **Protein ID** | **Homology (%)** | **Protein ID** | **Homology (%)** | **Protein ID** | **Homology (%)** |
| **Type Ⅰ** | | | | | | | | | | | | | |
| *tolC* | TolC family outer membrane protein | JHW38_20705 | QQP95623.1 | QQQ00452.1 | 82 | WP_096380486.1 | 85 | WP_057946311.1 | 81 | WP_057920732.1 | 79 | WP_057919129.1 | 75 |
| *hlyB* | type I secretion system permease/ATPase | JHW38_07240 | QQP97798.1 | NA | NA | WP_172437195.1 | 85 | NA | NA | NA | NA | WP_057917536.1 | 88 |
| *hlyD* | HlyD family type I secretion periplasmic adaptor subunit | JHW38_07235 | QQP97797.1 | NA | NA | WP_096377602.1 | 75 | NA | NA | NA | NA | WP_057917537.1 | 80 |
| **Type II** | | | | | | | | | | | | | |
| *gspD* | type II secretion system secretin GspD | JHW38_11525 | QQP98563.1 | QQQ02405.1 | 86 | WP_096376900.1 | 89 | WP_057948847.1 | 86 | WP_051547087.1 | 84 | WP_082647699.1 | 80 |
| *gspE* | type II secretion system ATPase GspE | JHW38_11575 | QQP98571.1 | QQQ03815.1 | 98 | WP_096383090.1 | 97 | WP_057948852.1 | 97 | WP_057923653.1 | 92 | WP_057919920.1 | 93 |
| *gspF* | type II secretion system F family protein | JHW38_11570 | QQP98570.1 | QQQ02399.1 | 95 | WP_096376894.1 | 97 | WP_057948850.1 | 95 | WP_057922480.1 | 94 | WP_057916902.1 | 92 |
| *gspG* | type II secretion system major pseudopilin GspG | JHW38_11565 | QQP98569.1 | QQQ02400.1 | 97 | WP_096376895.1 | 97 | WP_057948849.1 | 97 | WP_046659318.1 | 87 | WP_057916903.1 | 93 |
| *gspH* | GspH/FimT family pseudopilin | JHW38_11560 | QQP98568.1 | QQQ02401.1 | 92 | WP_074863290.1 | 93 | WP_082644871.1 | 90 | WP_082648914.1 | 90 | WP_082638231.1 | 86 |
| *gspI* | prepilin-type N-terminal cleavage/methylation domain-containing protein | JHW38_11555 | QQP98567.1 | QQQ02402.1 | 82 | WP_096376896.1 | 87 | WP_082644644.1 | 82 | WP_082648667.1 | 86 | WP_082647697.1 | 82 |
| *gspJ* | general secretion pathway protein GspJ | JHW38_11550 | QQP98566.1 | QQQ03816.1 | 92 | WP_096376897.1 | 93 | WP_057950262.1 | 92 | WP_057923651.1 | 84 | WP_057919923.1 | 89 |
| *gspK* | general secretion pathway protein GspK | JHW38_11545 | QQP98871.1 | QQQ03817.1 | 94 | WP_096383093.1 | 98 | WP_057950261.1 | 94 | WP_057922478.1 | 84 | WP_082647698.1 | 82 |
| *gspL* | PilN domain-containing protein | JHW38_11540 | QQP95730.1 | QQP98565.1 | 97 | WP_096376898.1 | 89 | WP_057946405.1 | 97 | WP_057920793.1 | 90 | WP_057919050.1 | 91 |
| *gspM* | general secretion pathway protein GspM | JHW38_11535 | QQP98870.1 | QQQ03818.1 | 94 | WP_096383097.1 | 96 | WP_082644870.1 | 93 | WP_036105013.1 | 87 | WP_082639047.1 | 87 |
| **Type ⅡI** | | | | | | | | | | | | | |
| *sctJ* | type III secretion inner membrane ring lipoprotein SctJ | JHW38_15620 | QQP94678.1 | QQQ01597.1 | 95 | WP_096382568.1 | 98 | WP_057949560.1 | 95 | WP_056109264.1 | 91 | WP_057919489.1 | 91 |
| *sctL* | hypothetical protein | JHW38_15610 | QQP94676.1 | QQQ01599.1 | 91 | WP_074871121.1 | 92 | WP_057949558.1 | 91 | WP_057921015.1 | 90 | WP_057919487.1 | 88 |
| *sctQ* | type III secretion system cytoplasmic ring protein SctQ | JHW38_15660 | QQP94686.1 | QQQ01588.1 | 84 | WP_096382596.1 | 92 | WP_057949569.1 | 84 | WP_036113395.1 | 74 | WP_057919494.1 | 64 |
| *sctS* | type III secretion system export apparatus subunit SctS | JHW38_15675 | QQP94687.1 | QQQ03780.1 | 96 | WP_074871139.1 | 98 | WP_101943760.1 | 95 | WP_036113388.1 | 97 | WP_057919495.1 | 93 |
| *sctT* | type III secretion system export apparatus subunit SctT | JHW38_15680 | QQP94688.1 | QQQ01587.1 | 95 | WP_096382601.1 | 98 | WP_057949571.1 | 95 | WP_036113385.1 | 85 | WP_036149407.1 | 87 |
| *sctU* | type III secretion system export apparatus subunit SctU | JHW38_15690 | QQP94690.1 | QQQ01586.1 | 94 | WP_096382604.1 | 95 | WP_057949572.1 | 94 | WP_057920381.1 | 88 | WP_051885283.1 | 88 |
| *sctV* | type III secretion system export apparatus subunit SctV | JHW38_15640 | QQP94682.1 | QQQ01593.1 | 95 | WP_096382578.1 | 96 | WP_057949563.1 | 95 | WP_082648235.1 | 90 | WP_057919491.1 | 90 |
| *yscN* | FliI/YscN family ATPase | JHW38_15605 | QQP94675.1 | QQQ01600.1 | 97 | WP_083382879.1 | 96 | WP_078997020.1 | 97 | WP_082124656.1 | 94 | WP_082639035.1 | 92 |
| *hrpA* | ATP-dependent RNA helicase HrpA | JHW38_10655 | QQP98400.1 | QQQ02538.1 | 93 | WP_096377021.1 | 94 | WP_057948691.1 | 93 | WP_057922400.1 | 84 | WP_057919008.1 | 85 |
| *hrpB* | ATP-dependent helicase HrpB | JHW38_16680 | QQP94875.1 | QQQ01384.1 | 91 | WP_096382752.1 | 92 | WP_057949751.1 | 91 | WP_057923337.1 | 89 | WP_057919800.1 | 90 |
| **Type Ⅳ** | | | | | | | | | | | | | |
| *virB1* | lytic transglycosylase domain-containing protein | JHW38_05095 | QQP98785.1 | QQQ03906.1 | 71 | WP_096377788.1 | 75 | WP_175429367.1 | 69 | WP_187313406.1 | 63 | WP_057917813.1 | 63 |
| *virB2* | TrbC/VirB2 family protein | JHW38_05090 | QQP97415.1 | QQQ03485.1 | 77 | WP_074869658.1 | 76 | WP_057947836.1 | 77 | WP_046656482.1 | 64 | WP_057917814.1 | 62 |
| *virB3* | VirB3 family type IV secretion system protein | JHW38_05085 | QQP97414.1 | QQQ03486.1 | 87 | WP_096377789.1 | 90 | WP_057947835.1 | 85 | WP_036103025.1 | 70 | WP_081930610.1 | 71 |
| *virB4* | VirB4 family type IV secretion/conjugal transfer ATPase | JHW38_05080 | QQQ03487.1 | QQQ03487.1 | 94 | WP_096377790.1 | 94 | WP_057947834.1 | 94 | WP_036103023.1 | 88 | WP_057917815.1 | 86 |
| *virB6* | type IV secretion system protein | JHW38_01705 | QQP96798.1 | QQQ03490.1 | 37 | WP_096377793.1 | 66 | WP_057945756.1 | 68 | WP_082648475.1 | 60 | WP_057919638.1 | 62 |
| *virB8* | type IV secretion system protein | JHW38_05115 | QQP97419.1 | QQQ03481.1 | 75 | WP_096377784.1 | 83 | WP_082644500.1 | 75 | WP_057921494.1 | 75 | WP_057920036.1 | 73 |
| *virB9* | TrbG/VirB9 family P-type conjugative transfer protein | JHW38_05110 | QQP97418.1 | QQQ03482.1 | 95 | WP_096377785.1 | 96 | WP_057947840.1 | 95 | WP_046656480.1 | 91 | WP_031370737.1 | 87 |
| *virB10* | TrbI/VirB10 family protein | JHW38_05105 | QQP97417.1 | QQQ03483.1 | 78 | WP_096377786.1 | 78 | WP_057947839.1 | 78 | WP_036103031.1 | 65 | WP_057917812.1 | 62 |
| *virB11* | P-type DNA transfer ATPase VirB11 | JHW38_05100 | QQP97416.1 | QQQ03484.1 | 95 | WP_096377787.1 | 98 | WP_057947838.1 | 95 | WP_036103029.1 | 91 | WP_031370739.1 | 89 |
| *virD4* | type IV secretory system conjugative DNA transfer family protein | JHW38_05005 | QQP98783.1 | QQQ03908.1 | 95 | WP_172437371.1 | 94 | WP_082644845.1 | 95 | WP_082124713.1 | 90 | WP_082639006.1 | 87 |
| **Type Ⅵ** | | | | | | | | | | | | | |
| *tssA* | type VI secretion system protein TssA | NA | NA | QQQ03700.1 | NA | NA | NA | WP_057947661.1 | NA | NA | NA | WP_057918151.1 | NA |
| *tssA* | type VI secretion system protein TssA | NA | NA | QQQ00764.1 | 100 | NA | NA | WP_057946032.1 | 99 | NA | NA | NA | NA |
| *tagF* | type VI secretion system-associated protein TagF | NA | NA | QQQ03703.1 | NA | NA | NA | WP_057947658.1 | NA | NA | NA | NA | NA |
| *tagF* | type VI secretion system-associated protein TagF | NA | NA | QQQ04050.1 | NA | NA | NA | WP_057949915.1 | NA | NA | NA | NA | NA |
| *tssM* | type VI secretion system membrane subunit TssM | NA | NA | QQQ00767.1 | NA | NA | NA | WP_082644264.1 | NA | NA | NA | NA | NA |
| *tssM* | type VI secretion system membrane subunit TssM | NA | NA | QQQ03919.1 | NA | NA | NA | WP_057950109.1 | NA | NA | NA | NA | NA |
| *tssL* | type VI secretion system protein TssL, long form | NA | NA | QQQ03704.1 | NA | NA | NA | WP_057947657.1 | NA | NA | NA | NA | NA |
| *tssK* | type VI secretion system baseplate subunit TssK | NA | NA | QQQ03705.1 | NA | NA | NA | WP_057947656.1 | NA | NA | NA | NA | NA |
| *tssK* | type VI secretion system baseplate subunit TssK | NA | NA | QQQ04047.1 | NA | NA | NA | WP_057946040.1 | NA | NA | NA | NA | NA |
| *tagH* | type VI secretion system-associated FHA domain protein TagH | NA | NA | QQQ03706.1 | NA | NA | NA | WP_082644481.1 | NA | NA | NA | NA | NA |
| *tssI* | type VI secretion system tip protein VgrG | NA | NA | QQQ03708.1 | NA | NA | NA | WP_057947653.1 | NA | NA | NA | NA | NA |
| *vgrG* | type VI secretion system tip protein VgrG | NA | NA | QQQ04048.1 | NA | NA | NA | WP_082644797.1 | NA | NA | NA | NA | NA |
| *vgrG* | type VI secretion system tip protein VgrG | NA | NA | QQQ04049.1 | NA | NA | NA | WP_057946033.1 | NA | NA | NA | NA | NA |
| *tssH* | type VI secretion system ATPase TssH | NA | NA | QQQ03709.1 | NA | NA | NA | WP_057946048.1 | NA | NA | NA | NA | NA |
| *tssH* | type VI secretion system ATPase TssH | NA | NA | QQQ00748.1 | NA | NA | NA | WP_057947651.1 | NA | NA | NA | NA | NA |
| *tssG* | type VI secretion system baseplate subunit TssG | NA | NA | QQQ03710.1 | NA | NA | NA | WP_082644480.1 | NA | NA | NA | NA | NA |
| *tssG* | type VI secretion system baseplate subunit TssG | NA | NA | QQQ00749.1 | NA | NA | NA | WP_057946047.1 | NA | NA | NA | NA | NA |
| *tssF* | type VI secretion system baseplate subunit TssF | NA | NA | QQQ03711.1 | NA | NA | NA | WP_057947650.1 | NA | NA | NA | NA | NA |
| *tssF* | type VI secretion system baseplate subunit TssF | NA | NA | QQQ00750.1 | NA | NA | NA | WP_057946046.1 | NA | NA | NA | NA | NA |
| *tssE* | type VI secretion system baseplate subunit TssE | NA | NA | QQQ03712.1 | NA | NA | NA | WP_057947649.1 | NA | NA | NA | NA | NA |
| *tssE* | type VI secretion system baseplate subunit TssE | NA | NA | QQQ00751.1 | NA | NA | NA | WP_057946045.1 | NA | NA | NA | NA | NA |
| *Hcp* | type VI secretion system tube protein Hcp | NA | NA | QQQ03714.1 | NA | NA | NA | WP_057947647.1 | NA | NA | NA | NA | NA |
| *Hcp* | type VI secretion system tube protein Hcp | NA | NA | QQQ00752.1 | NA | NA | NA | WP_057946044.1 | NA | NA | NA | NA | NA |
| *tssC* | type VI secretion system contractile sheath large subunit | NA | NA | QQQ03715.1 | NA | NA | NA | WP_057947646.1 | NA | NA | NA | NA | NA |
| *tssC* | type VI secretion system contractile sheath large subunit | NA | NA | QQQ00753.1 | NA | NA | NA | WP_057946043.1 | NA | NA | NA | NA | NA |
| *tssB* | type VI secretion system contractile sheath small subunit | NA | NA | QQQ03716.1 | NA | NA | NA | WP_057947645.1 | NA | NA | NA | NA | NA |
| *tssB* | type VI secretion system contractile sheath small subunit | NA | NA | QQQ00754.1 | NA | NA | NA | WP_057946042.1 | NA | NA | NA | NA | NA |
| *tssJ* | type VI secretion system lipoprotein TssJ | NA | NA | QQQ00755.1 | NA | NA | NA | WP_078998025.1 | NA | NA | NA | NA | NA |
| *icmH* | type IVB secretion system protein IcmH/DotU | NA | NA | QQQ00756.1 | NA | NA | NA | WP_057946039.1 | NA | NA | NA | NA | NA |
| **Sec-SRP** | | | | | | | | | | | | | |
| *secA* | preprotein translocase subunit SecA | JHW38_11275 | QQP98517.1 | QQQ02449.1 | 97 | WP_096376935.1 | 97 | WP_057948802.1 | 97 | WP_057922451.1 | 93 | WP_057916944.1 | 91 |
| *secB* | protein-export chaperone SecB | JHW38_18100 | QQP95137.1 | QQQ01663.1 | 92 | WP_074871611.1 | 96 | WP_057949501.1 | 91 | WP_036107609.1 | 91 | WP_031372765.1 | 89 |
| *secD* | protein translocase subunit SecD | JHW38_08420 | QQP98012.1 | QQQ02939.1 | 92 | WP_096377314.1 | 94 | WP_057948346.1 | 91 | WP_057921182.1 | 85 | WP_057917406.1 | 81 |
| *secE* | preprotein translocase subunit SecE | JHW38_09795 | QQP98257.1 | QQQ02682.1 | 93 | WP_074873738.1 | 95 | WP_057948567.1 | 93 | WP_036115412.1 | 87 | WP_057918904.1 | 84 |
| *secF* | protein translocase subunit SecF | JHW38_08415 | QQP98011.1 | QQQ02940.1 | 93 | WP_096377315.1 | 94 | WP_057948345.1 | 93 | WP_036101997.1 | 88 | WP_057917407.1 | 88 |
| *secG* | preprotein translocase subunit SecG | JHW38_02095 | QQP96870.1 | QQP99329.1 | 95 | WP_096378844.1 | 96 | WP_057947325.1 | 95 | WP_046656794.1 | 66 | WP_057918251.1 | 63 |
| *secY* | preprotein translocase subunit SecY | JHW38_09630 | QQP98226.1 | QQQ02714.1 | 99 | WP_074872155.1 | 99 | WP_057948537.1 | 99 | WP_036111756.1 | 95 | WP_031373816.1 | 96 |
| *yajC* | preprotein translocase subunit YajC | JHW38_08425 | QQP98013.1 | QQQ02938.1 | 84 | WP_074865736.1 | 95 | WP_057948347.1 | 84 | WP_036101993.1 | 87 | WP_031372943.1 | 81 |
| *yidC* | membrane protein insertase YidC | JHW38_16585 | QQP94856.1 | QQQ01404.1 | 97 | WP_096382816.1 | 91 | WP_057949735.1 | 98 | WP_057923352.1 | 81 | WP_057919817.1 | 81 |
| *ftsY* | signal recognition particle-docking protein FtsY | JHW38_08515 | QQP98031.1 | QQQ02920.1 | 74 | WP_096377302.1 | 97 | WP_057948362.1 | 74 | WP_057921172.1 | 91 | WP_057917394.1 | 74 |
| *ffh* | signal recognition particle protein | JHW38_07155 | QQP97781.1 | QQQ03137.1 | 97 | WP_096377478.1 | 99 | WP_057948157.1 | 97 | WP_057921281.1 | 95 | WP_057917550.1 | 95 |
| **Twin arginine targeting（Tet）** | | | | | | | | | | | | | |
| *tatA* | Sec-independent protein translocase subunit TatA | JHW38_17705 | QQP95062.1 | QQQ01212.1 | 92 | WP_074871763.1 | 72 | WP_057945811.1 | 92 | WP_036105095.1 | 68 | WP_031370598.1 | 58 |
| *tatB* | Sec-independent protein translocase protein TatB | JHW38_17710 | QQP95063.1 | QQQ01211.1 | 72 | WP_096376401.1 | 72 | WP_082644228.1 | 72 | WP_082648767.1 | 59 | WP_057916142.1 | 61 |
| *tatC* | twin-arginine translocase subunit TatC | JHW38_17715 | QQP95064.1 | QQQ01210.1 | 96 | WP_096376402.1 | 95 | WP_057945812.1 | 96 | WP_057922979.1 | 88 | WP_057916143.1 | 88 |

NA = not available.
