# Supplementary material for: Comparative genomics provides insights into the potential biocontrol mechanism of two Lysobacter enzymogenes strains with distinct antagonistic activities
Source: Front Microbiol. 2022 Aug 11;13:966986. doi: 10.3389/fmicb.2022.966986 (PMC9410377; doi:10.3389/fmicb.2022.966986)
Supplement: Supplementary file 4 [file Table_6.DOCX]

**Supplementary Table 6** Homology analysis of flagellar biosynthesis, Type IV pilus and chemotaxis in *Lysobacter enzymogenes* CX03, CX06 and other representative *Lysobacter* strains.

| **Strain** |  | ***L. enzymogenes* CX03** | | ***L. enzymogenes* CX06** | | ***L. enzymogenes* M497-1** | | ***L. enzymogenes* C3** | | ***L. capsici* 55** | | ***L. antibioticus* 76** | |
| --- | --- | --- | --- | --- | --- | --- | --- | --- | --- | --- | --- | --- | --- |
| **Genes** | **Product Definition** | **Locus Tag** | **Protein ID** | **Protein ID** | **Homology (%)** | **Protein ID** | **Homology (%)** | **Protein ID** | **Homology (%)** | **Protein ID** | **Homology (%)** | **Protein ID** | **Homology (%)** |
| **Basalbody/Hook** | | | | | | | | | | | | | |
| *fliE* | flagellar hook-basal body complex protein FliE | JHW38_13700 | QQP94326.1 | QQQ02011.1 | 96 | WP_096377175.1 | 44 | WP_197414772.1 | 90 | WP_036101681.1 | 39 | NA | NA |
| *fliF* | flagellar M-ring protein FliF | JHW38_13705 | QQP94327.1 | QQQ02010.1 | 88 | WP_096377176.1 | 35 | WP_057949201.1 | 86 | WP_057921013.1 | 34 | NA | NA |
| *fliH* | flagellar assembly protein FliH | JHW38_15610 | QQP94676.1 | QQQ01599.1 | 91 | WP_096377178.1 | 92 | WP_057949558.1 | 90 | WP_057921015.1 | 90 | WP_057919487.1 | 88 |
| *fliI* | flagellum-specific ATP synthase | JHW38_15605 | QQP94675.1 | QQQ01600.1 | 97 | WP_096377179.1 | 96 | WP_078997020.1 | 97 | WP_036101687.1 | 94 | WP_082639035.1 | 92 |
| *fliM* | FliM family flagellar motor switch protein | JHW38_13745 | QQP94335.1 | QQQ02002.1 | 81 | WP_096377185.1 | 30 | WP_057949210.1 | 80 | NA | NA | NA | NA |
| *fliN* | flagellar motor switch protein FliN | JHW38_13750 | QQP94336.1 | QQQ02001.1 | 100 | WP_096377185.1 | 41 | WP_057949210.1 | 87 | WP_082648374.1 | 40 | NA | NA |
| *fliO* | flagellar biosynthetic protein FliO | JHW38_15645 | QQP94683.1 | QQQ01592.1 | 69 | WP_145960220.1 | 84 | WP_057949564.1 | 74 | WP_057920383.1 | 77 | WP_057919492.1 | 73 |
| *fliP* | flagellar biosynthesis protein FliP | JHW38_13760 | QQP94338.1 | QQQ01999.1 | 96 | WP_096377187.1 | 48 | WP_123646598.1 | 96 | WP_096418215.1 | 50 | NA | NA |
| *fliQ* | flagellar type III secretion system protein FliQ | JHW38_13645 | QQP94315.1 | QQQ02022.1 | 94 | WP_096377167.1 | 46 | WP_057949191.1 | 94 | WP_036101654.1 | 47 | NA | NA |
| *fliR* | flagellar biosynthesis protein FliR | JHW38_13650 | QQP94316.1 | QQQ02021.1 | 95 | WP_096377168.1 | 33 | WP_057949192.1 | 96 | WP_148650709.1 | 34 | NA | NA |
| *flgG* | flagellar hook-basal body protein | JHW38_13670 | QQP94320.1 | QQQ02017.1 | 91 | WP_057949195.1 | 91 | WP_096377171.1 | 35 | WP_057921010.1 | 33 | NA | NA |
| *flgD* | flagellar hook capping protein | JHW38_13735 | QQP94333.1 | NA | NA | WP_096377182.1 | 40 | WP_057949207.1 | 93 | WP_036101693.1 | 39 | NA | NA |
| *flgE* | flagellar hook-basal body complex protein | JHW38_13740 | QQP94334.1 | QQQ02003.1 | 77 | WP_096377183.1 | 30 | WP_057949208.1 | 77 | WP_057921018.1 | 27 | NA | NA |
| *flgH* | flagellar basal body L-ring protein FlgH | JHW38_13680 | QQP94322.1 | QQQ02015.1 | 93 | WP_096383190.1 | 40 | WP_057949198.1 | 94 | WP_057921010.1 | 40 | NA | NA |
| *flgI* | flagellar basal body P-ring protein FlgI | JHW38_13685 | QQP94323.1 | QQQ02014.1 | 91 | WP_096383194.1 | 44 | WP_057950305.1 | 91 | WP_036101669.1 | 45 | NA | NA |
| **Type IV pilus** | | | | | | | | | | | | | |
| *pilW* | type IV pilus biogenesis/stability protein PilW | JHW38_05555 | QQP97489.1 | QQQ03415.1 | 83 | WP_083382686.1 | 84 | WP_057947901.1 | 82 | WP_051546885.1 | 66 | WP_082647817.1 | 63 |
| *pilE* | type IV pilin protein PiLE | JHW38_08730 | QQP98066.1 | QQQ03849.1 | 47 | WP_096377269.1 | 57 | WP_057950210.1 | 46 | WP_046656007.1 | 61 | WP_057917368.1 | 62 |
| *pilC* | pilus assembly protein PilC | JHW38_08735 | QQP98067.1 | QQQ02837.1 | 35 | WP_096377268.1 | 57 | WP_057948392.1 | 36 | WP_057921152.1 | 66 | WP_057917367.1 | 69 |
| *pilV* | type IV pilus modification protein PilV | JHW38_08750 | QQP98069.1 | QQQ02834.1 | 44 | WP_172437176.1 | 56 | WP_082644574.1 | 44 | WP_148650265.1 | 68 | WP_057917364.1 | 67 |
| *pilG* | twitching motility response regulator PilG | JHW38_10540 | QQP98378.1 | QQQ02553.1 | 99 | WP_031372856.1 | 100 | WP_057948678.1 | 99 | WP_036112825.1 | 99 | WP_031372856.1 | 100 |
| *pilA* | type IV pilus assembly protein PilA | JHW38_11190 | QQP98501.1 | QQQ02456.1 | 61 | WP_096376896.1 | 72 | WP_231784057.1 | 58 | WP_057922445.1 | 66 | WP_237051792.1 | 57 |
| *pilB* | type IV-A pilus assembly ATPase PilB | JHW38_11250 | QQP98512.1 | QQQ02454.1 | 90 | WP_096376940.1 | 97 | WP_082644635.1 | 93 | WP_057922447.1 | 92 | WP_057916949.1 | 89 |
| *pilD* | prepilin peptidase | JHW38_11260 | QQP98514.1 | QQQ02452.1 | 95 | WP_096376938.1 | 95 | WP_057948799.1 | 95 | WP_036104586.1 | 89 | WP_057916947.1 | 88 |
| *pilU* | PilT/PilU family type 4a pilus ATPase | JHW38_11870 | QQP98628.1 | QQQ02339.1 | 99 | WP_096376847.1 | 99 | WP_057948907.1 | 99 | WP_036104874.1 | 96 | WP_057916856.1 | 96 |
| *pilM* | pilus assembly protein PilM | JHW38_21285 | QQP95729.1 | QQQ00348.1 | 99 | WP_074864544.1 | 99 | WP_057946404.1 | 99 | WP_036108732.1 | 97 | WP_057919051.1 | 98 |
| *pilO* | type 4a pilus biogenesis protein PilO | JHW38_21295 | QQP95731.1 | QQQ00346.1 | 94 | WP_096380069.1 | 96 | WP_057946406.1 | 94 | WP_057920794.1 | 87 | WP_057919049.1 | 87 |
| *pilP* | pilus assembly protein PilP | JHW38_21300 | QQP95732.1 | QQQ00345.1 | 91 | WP_074864541.1 | 93 | WP_057946407.1 | 91 | WP_057920795.1 | 89 | WP_057919048.1 | 87 |
| *pilQ* | type IV pilus secretin PilQ | JHW38_21305 | QQP95733.1 | QQQ00344.1 | 87 | WP_232518550.1 | 90 | WP_057949969.1 | 87 | WP_237051054.1 | 85 | WP_057920190.1 | 84 |
| *pilT* | type IV pilus twitching motility protein PilT | JHW38_22410 | QQP95934.1 | QQQ00162.1 | 100 | WP_074864167.1 | 99 | WP_057946565.1 | 99 | WP_036109068.1 | 98 | WP_031370959.1 | 98 |
| *fimT* | type IV pilus assembly protein FimT | JHW38_08755 | QQP98831.1 | QQQ02868.1 | 43 | WP_172437175.1 | 54 | WP_158229892.1 | 42 | WP_187313363.1 | 57 | WP_148649667.1 | 57 |
| **Global regulator** | | | | | | | | | | | | | |
| *rpoD* | RNA polymerase sigma factor RpoD | JHW38_18905 | QQP95289.1 | QQQ01020.1 | 97 | WP_096381557.1 | 98 | WP_057945983.1 | 97 | WP_057922831.1 | 93 | WP_057916321.1 | 92 |
| *rpoN* | RNA polymerase factor sigma-54 | JHW38_22535 | QQP95958.1 | QQQ00141.1 | 94 | WP_096379591.1 | 96 | WP_057946586.1 | 94 | WP_036102302.1 | 89 | WP_057917242.1 | 85 |
| sig | sigma-70 family RNA polymerase sigma factor | JHW38_13765 | QQP94339.1 | QQQ01982.1 | NA | WP_096377188.1 | 33 | WP_057946087.1 | 38 | WP_057921021.1 | 38 | NA | NA |
| **Chemotaxis genes** | | | | | | | | | | | | | |
| *cheW* | chemotaxis protein CheW | JHW38_10510 | QQP98373.1 | QQQ02558.1 | 92 | WP_074864420.1 | 89 | WP_057947546.1 | 93 | WP_057921628.1 | 82 | WP_057918986.1 | 79 |
| *pilJ* | methyl-accepting chemotaxis protein | JHW38_10525 | QQP98376.1 | QQQ02555.1 | 97 | WP_096377033.1 | 97 | WP_057948676.1 | 99 | WP_036112833.1 | 94 | WP_057918989.1 | 92 |
| *pilI* | purine-binding chemotaxis protein CheW | JHW38_10530 | QQP98860.1 | QQQ03828.1 | 97 | WP_074863320.1 | 93 | WP_057950245.1 | 96 | WP_036112830.1 | 82 | WP_057918990.1 | 82 |
| *cheB* | chemotaxis response regulator protein-glutamate methylesterase | JHW38_21665 | QQP95797.1 | QQP99088.1 | 85 | WP_096379850.1 | 89 | WP_057947545.1 | 86 | WP_057921632.1 | 63 | WP_057916594.1 | 67 |
| *wspD* | purine-binding chemotaxis protein CheW | JHW38_21675 | QQP95799.1 | NA | NA | WP_096379845.1 | 85 | WP_057947546.1 | 80 | WP_057921630.1 | 52 | WP_057916592.1 | 57 |
| *wspB* | purine-binding chemotaxis protein CheW | JHW38_21685 | QQP95801.1 | QQP99085.1 | 86 | WP_074864420.1 | 88 | WP_057947548.1 | 87 | WP_057921628.1 | 59 | WP_057916590.1 | 56 |
| *wspA* | methyl-accepting chemotaxis protein | JHW38_21690 | QQP95802.1 | QQP99084.1 | 86 | WP_096379841.1 | 89 | WP_057947549.1 | 86 | LC55x_2662 | 67 | WP_057916589.1 | 69 |

NA = not available.
