# Supplementary material for: Comparative genomics provides insights into the potential biocontrol mechanism of two Lysobacter enzymogenes strains with distinct antagonistic activities
Source: Front Microbiol. 2022 Aug 11;13:966986. doi: 10.3389/fmicb.2022.966986 (PMC9410377; doi:10.3389/fmicb.2022.966986)
Supplement: Supplementary file 5 [file Table_7.DOCX]

**Supplementary Table 7** Homology analysis of in two-component system in *Lysobacter enzymogenes* CX03, CX06 and other representative *Lysobacter* strains.

| **Strain** |  | ***L. enzymogenes* CX03** | | ***L. enzymogenes* CX06** | | ***L. enzymogenes* M497-1** | | ***L. enzymogenes* C3** | | ***L. capsici* 55** | | ***L. antibioticus* 76** | |
| --- | --- | --- | --- | --- | --- | --- | --- | --- | --- | --- | --- | --- | --- |
| **Genes** | **Product Definition** | **Locus Tag** | **Protein ID** | **Protein ID** | **Homology (%)** | **Protein ID** | **Homology (%)** | **Protein ID** | **Homology (%)** | **Protein ID** | **Homology (%)** | **Protein ID** | **Homology (%)** |
| **Two-component system** | | | | | | | | | | | | | |
| **OmpR family** | | | | | | | | | | | | | |
| *phoR* | phosphate regulon sensor histidine kinase PhoR | JHW38_24545 | QQP96334.1 | QQP96334.1 | 97 | WP_074863361.1 | 97 | WP_057946944.1 | 97 | WP_057922087.1 | 94 | WP_057918648.1 | 94 |
| *phoB* | phosphate regulon transcriptional regulator PhoB | JHW38_24550 | QQP96335.1 | QQP99761.1 | 100 | WP_057946945.1 | 100 | WP_057946945.1 | 100 | WP_036112073.1 | 98 | WP_031373561.1 | 98 |
| *pstS* | phosphate ABC transporter substrate-binding protein PstS | JHW38_07395 | QQP97826.1 | QQQ03092.1 | 96 | WP_096377432.1 | 99 | WP_078998666.1 | 95 | WP_148650298.1 | 91 | WP_057917515.1 | 93 |
| *pstS* | phosphate ABC transporter substrate-binding protein PstS | JHW38_07400 | QQP97827.1 | QQQ03091.1 | 94 | WP_096377431.1 | 95 | WP_057948200.1 | 95 | WP_046658918.1 | 91 | WP_057917514.1 | 90 |
| *phoQ* | two-component sensor histidine kinase | JHW38_12265 | QQP98701.1 | QQQ02273.1 | 95 | WP_074862176.1 | 97 | WP_057948977.1 | 95 | WP_082648919.1 | 93 | WP_057916805.1 | 91 |
| *phoP* | response regulator transcription factor | JHW38_12270 | QQP98702.1 | QQQ02272.1 | 100 | WP_031372686.1 | 100 | WP_031372686.1 | 100 | WP_036106626.1 | 99 | WP_031372686.1 | 100 |
| *creC* | two-component system sensor histidine kinase CreC | JHW38_24480 | QQP96321.1 | QQP99777.1 | 94 | WP_074863398.1 | 97 | WP_057946934.1 | 94 | WP_057922098.1 | 86 | WP_057918656.1 | 84 |
| *creB* | two-component system response regulator CreB | JHW38_24475 | QQP96320.1 | QQP99778.1 | 90 | WP_145960236.1 | 93 | WP_057946933.1 | 91 | WP_036109243.1 | 88 | WP_057918657.1 | 91 |
| *cusS* | heavy metal sensor histidine kinase | JHW38_25205 | QQP96454.1 | QQQ03962.1 | 84 | WP_096383590.1 | 85 | WP_078997397.1 | 83 | WP_057922009.1 | 76 | WP_057918562.1 | 77 |
| *cusR* | heavy metal response regulator transcription factor | JHW38_25200 | QQP96453.1 | QQP99652.1 | 96 | WP_074867468.1 | 97 | WP_057947045.1 | 96 | WP_046657101.1 | 92 | WP_057918563.1 | 89 |
| *kdpD* | sensor histidine kinase KdpD | JHW38_06945 | QQP97744.1 | QQQ03182.1 | 96 | WP_096377524.1 | 96 | WP_057948116.1 | 96 | WP_057921306.1 | 90 | WP_057917569.1 | 90 |
| *kdpE* | response regulator | JHW38_06950 | QQP97745.1 | QQQ03181.1 | 94 | WP_096377523.1 | 95 | WP_057948117.1 | 94 | WP_057921305.1 | 94 | WP_057917568.1 | 93 |
| *tctE* | sensor histidine kinase N-terminal domain-containing protein | JHW38_14400 | QQP94458.1 | QQQ01877.1 | 95 | WP_096376512.1 | 94 | WP_057949309.1 | 95 | WP_057923401.1 | 81 | WP_057920225.1 | 82 |
| *tctD* | response regulator transcription factor | JHW38_14405 | QQP94459.1 | QQQ01876.1 | 97 | WP_096376511.1 | 96 | WP_057949310.1 | 97 | WP_036102564.1 | 91 | WP_057919297.1 | 84 |
| **LytTR family** | | | | | | | | | | | | | |
| *algZ* | histidine kinase | JHW38_13285 | QQP94247.1 | QQQ02079.1 | 94 | WP_074861707.1 | 96 | WP_082644678.1 | 94 | WP_082124743.1 | 89 | WP_057916665.1 | 90 |
| *algR* | response regulator transcription factor | JHW38_13280 | QQP94246.1 | QQQ02080.1 | 98 | WP_074861708.1 | 98 | WP_057949142.1 | 98 | WP_036114843.1 | 93 | WP_031372561.1 | 93 |
| *natA* | ATP-binding cassette domain-containing protein | JHW38_16630 | QQP94865.1 | QQQ01395.1 | 96 | WP_096382789.1 | 96 | WP_057949744.1 | 96 | WP_036103264.1 | 94 | WP_057919811.1 | 92 |
| *natB* | ABC transporter permease | JHW38_16625 | QQP94864.1 | QQQ01396.1 | 95 | WP_096382793.1 | 96 | WP_057949743.1 | 95 | WP_057923344.1 | 84 | WP_057919812.1 | 85 |
| **narL family** | | | | | | | | | | | | | |
| *desR* | response regulator transcription factor | JHW38_08440 | QQP98016.1 | QQQ02935.1 | 100 | WP_074870337.1 | 99 | WP_057948350.1 | 100 | WP_036101987.1 | 99 | WP_031372940.1 | 97 |
| *desK* | sensor histidine kinase | JHW38_08445 | QQP98017.1 | QQQ02934.1 | 96 | WP_145960011.1 | 98 | WP_057948351.1 | 96 | WP_046656056.1 | 82 | WP_057917404.1 | 74 |
| *rcsC* | transporter substrate-binding domain-containing protein | JHW38_04030 | QQP97226.1 | NA | NA | WP_172437223.1 | 77 | WP_057947691.1 | 68 | WP_057921692.1 | 51 | NA | NA |
| *rcsB* | captular synthesis response regulator | JHW38_04035 | QQP98769.1 | NA | NA | WP_074870337.1 | 87 | WP_057950116.1 | 76 | WP_046656657.1 | 71 | NA | NA |
| *eal* | EAL domain-containing response regulator | JHW38_15850 | QQP94718.1 | QQQ01556.1 | 85 | WP_172437350.1 | 88 | WP_057949600.1 | 85 | WP_046659686.1 | 82 | WP_187308427.1 | 79 |
| *evgS* | response regulator | JHW38_15855 | QQP94719.1 | QQQ01555.1 | 84 | WP_096382706.1 | 89 | WP_057949601.1 | 84 | WP_057920364.1 | 72 | WP_057919520.1 | 71 |
| **ntrC family** | | | | | | | | | | | | | |
| *ntrC* | nitrogen regulation protein NR(I) | JHW38_18030 | QQP95124.1 | QQQ01649.1 | 92 | WP_096376446.1 | 94 | WP_057949514.1 | 92 | WP_057920421.1 | 89 | WP_057919449.1 | 88 |
| *ntrB* | PAS domain-containing sensor histidine kinase | JHW38_18025 | QQP95123.1 | QQQ01648.1 | 95 | WP_096376445.1 | 95 | WP_057949515.1 | 95 | WP_057920420.1 | 91 | WP_057919450.1 | 87 |
| *pilS* | PAS domain-containing sensor histidine kinase | JHW38_11180 | QQP98499.1 | QQQ02458.1 | 96 | WP_096376445.1 | 97 | WP_057948793.1 | 96 | WP_057922444.1 | 90 | WP_057919450.1 | 89 |
| *pilR* | sigma-54-dependent Fis family transcriptional regulator | JHW38_11185 | QQP98500.1 | QQQ02457.1 | 96 | WP_074862710.1 | 97 | WP_057948794.1 | 97 | WP_036104561.1 | 92 | WP_057916960.1 | 92 |
| *ntrY* | nitrogen regulation sensor histidine | JHW38_20115 | QQP95518.1 | QQQ00552.1 | 91 | WP_096380811.1 | 97 | WP_096380811.1 | 97 | WP_096418138.1 | 85 | WP_057919205.1 | 89 |
| *dctD* | sigma-54-dependent Fis family transcriptional regulator | JHW38_20120 | QQP95519.1 | QQQ00551.1 | 92 | WP_096380807.1 | 95 | WP_057946223.1 | 95 | WP_036108922.1 | 87 | WP_057919204.1 | 86 |
| **Chemotaxis family** | | | | | | | | | | | | | |
| *cheA* | Hpt domain-containing protein | JHW38_10520 | QQP98375.1 | QQQ02556.1 | 88 | WP_096377038.1 | 92 | WP_057948675.1 | 84 | WP_057922391.1 | 78 | WP_057918988.1 | 78 |
| *cheW* | chemotaxis protein CheW | JHW38_10510 | QQP98373.1 | QQQ02558.1 | 92 | WP_096377036.1 | 89 | WP_082644614.1 | 93 | WP_046657728.1 | 82 | WP_057918986.1 | 79 |
| *pilG* | twitching motility response regulator PilG | JHW38_10540 | QQP98378.1 | QQQ02553.1 | 99 | WP_031372856.1 | 100 | WP_057948678.1 | 99 | WP_036112825.1 | 100 | WP_031372856.1 | 99 |
| *pilH* | response regulator | JHW38_10535 | QQP98377.1 | QQQ02554.1 | 98 | WP_074862927.1 | 99 | WP_057948677.1 | 98 | WP_036112827.1 | 93 | WP_031372855.1 | 92 |
| *wspA* | methyl-accepting chemotaxis protein | JHW38_21690 | QQP95802.1 | QQP99084.1 | 86 | WP_096379841.1 | 89 | WP_057947549.1 | 86 | WP_057921627.1 | 67 | WP_057916589.1 | 69 |
| *wspB* | purine-binding chemotaxis protein CheW | JHW38_21685 | QQP95801.1 | QQP99085.1 | 86 | WP_074864420.1 | 88 | WP_057947548.1 | 87 | WP_057921628.1 | 59 | WP_057916590.1 | 56 |
| *wspE* | hybrid sensor histidine kinase/response regulator | JHW38_21670 | QQP95798.1 | QQQ03926.1 | 89 | WP_096379848.1 | 90 | WP_057950097.1 | 89 | WP_057921631.1 | 66 | WP_057916593.1 | 68 |
| *wspF* | chemotaxis response regulator protein-glutamate methylesterase | JHW38_21665 | QQP95797.1 | QQP99088.1 | 85 | WP_096379850.1 | 89 | WP_057947545.1 | 86 | WP_057921632.1 | 63 | WP_057916594.1 | 67 |
| **Other family** | | | | | | | | | | | | | |
| *rpfC* | response regulator | JHW38_02835 | QQP97009.1 | QQP99222.1 | 92 | WP_096378745.1 | 47 | WP_057947419.1 | 92 | WP_082648799.1 | 30 | WP_057918114.1 | 48 |
| *rpfG* | two-component system response regulator | JHW38_02830 | QQP97008.1 | QQP99223.1 | 99 | WP_096378746.1 | 97 | WP_057947418.1 | 99 | WP_082124289.1 | 97 | WP_031372280.1 | 95 |
| *regB* | HAMP domain-containing histidine kinase | JHW38_03230 | QQP97082.1 | QQP99154.1 | 97 | WP_198420086.1 | 97 | WP_057947489.1 | 97 | WP_057921605.1 | 89 | WP_057918039.1 | 84 |
| *regA* | response regulator transcription factor | JHW38_03225 | QQP97081.1 | QQP99155.1 | 94 | WP_096378691.1 | 96 | WP_057947488.1 | 97 | WP_082648501.1 | 91 | WP_057918040.1 | 92 |
| *regR* | response regulator | JHW38_02290 | QQP96904.1 | QQQ03944.1 | 94 | WP_096378819.1 | 92 | WP_057950076.1 | 92 | WP_046656770.1 | 81 | WP_057918220.1 | 82 |
| *regS* | HAMP domain-containing histidine kinase | JHW38_02285 | QQP96903.1 | QQP99292.1 | 93 | WP_145960086.1 | 94 | WP_057947359.1 | 92 | WP_057921805.1 | 82 | WP_057918221.1 | 81 |
| *epsA* | polysaccharide biosynthesis/export family protein | JHW38_02750 | QQP96995.1 | QQP99236.1 | 94 | WP_074868888.1 | 94 | WP_057947406.1 | 95 | WP_036107278.1 | 76 | WP_057918534.1 | 72 |
| *epsB* | polysaccharide biosynthesis tyrosine autokinase | JHW38_02735 | QQP96992.1 | QQP99238.1 | 92 | WP_096378760.1 | 94 | WP_057947405.1 | 92 | WP_057921625.1 | 74 | WP_057918536.1 | 70 |
| *tctD* | response regulator transcription factor | JHW38_10705 | QQP98410.1 | QQQ01876.1 | 61 | WP_096377014.1 | 98 | WP_057949310.1 | 61 | WP_046657745.1 | 90 | WP_057919297.1 | 61 |
| *tctE* | sensor histidine kinase, two-component system, OmpR family | JHW38_10710 | QQP98411.1 | QQQ01877.1 | 54 | WP_096377013.1 | 93 | WP_057949309.1 | 54 | WP_057922406.1 | 80 | WP_057920225.1 | 54 |
| *fecR* | FecR domain-containing protein | JHW38_20315 | QQP95549.1 | QQQ00518.1 | 84 | WP_074873428.1 | 85 | WP_057946256.1 | 85 | WP_046660060.1 | 68 | WP_057919175.1 | 71 |
| *pupR* | RNA polymerase sigma factor | JHW38_20310 | QQP98975.1 | QQQ04025.1 | 95 | WP_193830644.1 | 97 | WP_082644807.1 | 92 | WP_082124824.1 | 87 | WP_081930671.1 | 81 |

NA = not available.
