# Supplementary material for: Comparative genomics provides insights into the potential biocontrol mechanism of two Lysobacter enzymogenes strains with distinct antagonistic activities
Source: Front Microbiol. 2022 Aug 11;13:966986. doi: 10.3389/fmicb.2022.966986 (PMC9410377; doi:10.3389/fmicb.2022.966986)
Supplement: Supplementary file 6 [file Table_8.DOCX]

**Supplementary Table 8** Homology analysis of quorum sensing in *Lysobacter enzymogenes* CX03 and other representative *Lysobacter* strains.

| **Strain** |  | ***L. enzymogenes* CX03** | | ***L. enzymogenes* CX06** | | | ***L. enzymogenes* M497-1** | | | ***L. enzymogenes* C3** | | | ***L. capsici* 55** | | | ***L. antibioticus* 76** | |
| --- | --- | --- | --- | --- | --- | --- | --- | --- | --- | --- | --- | --- | --- | --- | --- | --- | --- |
| **Genes** | **Product Definition** | **Locus Tag** | **Protein ID** | **Protein ID** | **Homology (%)** | **Protein ID** | | **Homology (%)** | | **Protein ID** | **Homology (%)** | | **Protein ID** | **Homology (%)** | | **Protein ID** | **Homology (%)** |
| *Hfq* | RNA chaperone Hfq | JHW38_23910 | QQP96212.1 | QQP99881.1 | 100 | | WP_096379284.1 | | 99 | WP_057946835.1 | | 100 | WP_036114113.1 | | 98 | WP_031374342.1 | 95 |
| *trpE* | anthranilate synthase component I | JHW38_11990 | QQP98651.1 | QQQ02319.1 | 97 | | WP_074862308.1 | | 96 | WP_057948933.1 | | 97 | WP_046657879.1 | | 91 | WP_057916837.1 | 91 |
| *pabA* | aminodeoxychorismate/anthranilate synthase component II | JHW38_11965 | QQP98646.1 | QQQ02324.1 | 97 | | WP_074862318.1 | | 96 | WP_057948921.1 | | 97 | WP_057922530.1 | | 89 | WP_057916843.1 | 83 |
| *aroG* | 3-deoxy-7-phosphoheptulonate synthase class II | JHW38_09595 | QQP98219.1 | QQQ02721.1 | 98 | | WP_096377141.1 | | 98 | WP_057948530.1 | | 99 | WP_036111723.1 | | 96 | WP_057918895.1 | 96 |
| *blcC* | N-acyl homoserine lactonase family protein | JHW38_19295 | QQP95364.1 | NA | NA | | WP_096381307.1 | | 83 | NA | | NA | NA | | NA | WP_057917324.1 | 54 |
| *zmpA* | M4 family metallopeptidase | JHW38_16450 | QQP94829.1 | QQQ01327.1 | 43 | | WP_096376169.1 | | 91 | WP_057949712.1 | | 90 | WP_082648211.1 | | 81 | WP_187308446.1 | 77 |
| *zmpA* | M4 family metallopeptidase | JHW38_16990 | QQP94934.1 | QQQ01428.1 | 41 | | WP_096376285.1 | | 86 | WP_057949712.1 | | 41 | WP_082648211.1 | | 43 | WP_187308446.1 | 43 |
| *bapA* | DUF11 domain-containing protein | JHW38_09440 | QQP98190.1 | QQQ02751.1 | 89 | | WP_172437169.1 | | 93 | WP_197414744.1 | | 89 | WP_187313348.1 | | 84 | WP_057916779.1 | 53 |
| *bapA* | putative Ig domain-containing protein | JHW38_09370 | QQP98177.1 | QQQ02755.1 | 48 | | WP_096377162.1 | | 75 | WP_057948498.1 | | 67 | WP_057921006.1 | | 59 | WP_082647893.1 | 34 |
| *bapA* | putative Ig domain-containing protein | JHW38_01845 | QQP96823.1 | QQP99386.1 | 83 | | WP_172437263.1 | | 87 | WP_082644425.1 | | 83 | WP_187313216.1 | | 74 | WP_082647893.1 | 72 |
| *rpfB* | long-chain fatty acid--CoA ligase | JHW38_02845 | QQP97011.1 | QQP99220.1 | 93 | | WP_096378743.1 | | 96 | WP_057947421.1 | | 93 | WP_057921538.1 | | 88 | WP_057918112.1 | 88 |
| *rpfC* | response regulator | JHW38_15855 | QQP94719.1 | QQQ01555.1 | 84 | | WP_096382706.1 | | 89 | WP_057949601.1 | | 84 | WP_057920364.1 | | 72 | WP_057919520.1 | 71 |
| *rpfG* | two-component system response regulator | JHW38_02830 | QQP97008.1 | QQP99223.1 | 99 | | WP_096378746.1 | | 91 | WP_057947418.1 | | 99 | WP_082124289.1 | | 97 | WP_031372280.1 | 95 |
| *clp* | Crp/Fnr family transcriptional regulator | JHW38_09480 | QQP98197.1 | QQQ01988.1 | 29 | | WP_074861652.1 | | 28 | WP_197414776.1 | | 29 | WP_187313347.1 | | 83 | WP_081930655.1 | 28 |
| *crp* | cAMP-activated global transcriptional regulator CRP | JHW38_11935 | QQP98640.1 | QQQ02330.1 | 100 | | WP_031371492.1 | | 100 | WP_031371492.1 | | 100 | WP_082124483.1 | | 100 | WP_031371492.1 | 100 |
| *zur* | transcriptional repressor | JHW38_23195 | QQP96083.1 | QQQ00011.1 | 99 | | WP_096379427.1 | | 94 | WP_057946707.1 | | 98 | WP_036114650.1 | | 93 | WP_036149149.1 | 93 |
| *ribD* | bifunctional diaminohydroxyphosphoribosylaminopyrimidine deaminase/5-amino-6-(5-phosphoribosylamino)uracil reductase RibD | JHW38_11450 | QQP98548.1 | QQQ02419.1 | 94 | | WP_096376913.1 | | 94 | WP_057948832.1 | | 94 | WP_057922468.1 | | 89 | WP_057916918.1 | 90 |
| *toxl* | efflux transporter outer membrane subunit | JHW38_16110 | QQP94764.1 | QQQ01505.1 | 96 | | WP_096376276.1 | | 33 | WP_057949646.1 | | 96 | WP_057920326.1 | | 93 | WP_057919243.1 | 32 |
| *oprM* | efflux transporter outer membrane subunit | JHW38_13940 | QQP94371.1 | QQQ01968.1 | 92 | | WP_096376568.1 | | 95 | WP_057949238.1 | | 92 | WP_057920646.1 | | 84 | WP_057916101.1 | 33 |
| *oppA* | peptide ABC transporter substrate-binding protein | JHW38_14645 | QQP94503.1 | QQQ01037.1 | 91 | | WP_172437341.1 | | 94 | WP_175429092.1 | | 92 | WP_187313328.1 | | 85 | WP_148649834.1 | 86 |
| *oppB* | ABC transporter permease subunit | JHW38_14655 | QQP94505.1 | QQQ01039.1 | 95 | | WP_096381995.1 | | 97 | WP_057945968.1 | | 95 | NA | | NA | NA | NA |
| *oppC* | ABC transporter permease | JHW38_18265 | QQP95169.1 | QQQ01711.1 | 93 | | WP_172437118.1 | | 95 | WP_057949462.1 | | 93 | NA | | NA | NA | NA |
| *dpp* | ABC transporter ATP-binding protein | JHW38_18270 | QQP95170.1 | QQQ01712.1 | 89 | | WP_096376478.1 | | 92 | NA | | NA | WP_057922937.1 | | 81 | NA | NA |
| *yidC* | membrane protein insertase YidC | JHW38_16585 | QQP94856.1 | QQQ01404.1 | 97 | | WP_096382816.1 | | 91 | WP_057949735.1 | | 98 | WP_057923352.1 | | 81 | WP_057919817.1 | 81 |
| *sprE* | trypsin-like serine protease | JHW38_10790 | QQP98427.1 | QQP99518.1 | 65 | | WP_096383786.1 | | 94 | NA | | NA | WP_057921639.1 | | 75 | WP_082647920.1 | 43 |
| *bprV* | S8 family serine peptidase | JHW38_06050 | QQP97580.1 | QQQ02648.1 | 55 | | WP_096377113.1 | | 56 | WP_082644600.1 | | 56 | WP_082648634.1 | | 56 | NA | NA |
| *bprV* | S8 family serine peptidase | JHW38_10065 | QQP98292.1 | QQQ02650.1 | 58 | | WP_172437162.1 | | 67 | WP_138885234.1 | | 58 | NA | | NA | NA | NA |
| *bprV* | S8 family serine peptidase | JHW38_10070 | QQP98292.1 | QQQ02646.1 | 50 | | WP_172437162.1 | | 67 | WP_057948596.1 | | 50 | NA | | NA | NA | NA |
| *bprV* | S8 family serine peptidase | JHW38_01400 | QQP96739.1 | QQQ02965.1 | 42 | | WP_172437163.1 | | 32 | WP_057947195.1 | | 87 | WP_057923295.1 | | 66 | WP_057918376.1 | 79 |
| *bprV* | S8 family serine peptidase | JHW38_07315 | QQP97812.1 | QQQ03105.1 | 91 | | WP_172437188.1 | | 95 | WP_057948186.1 | | 91 | WP_187313371.1 | | 85 | WP_057917526.1 | 84 |
| *bprV* | S8 family serine peptidase | JHW38_04060 | QQP97231.1 | QQQ02965.1 | 31 | | WP_172437222.1 | | 92 | WP_057947023.1 | | 26 | WP_057923295.1 | | 30 | WP_057917939.1 | 73 |
| *expR* | S8 family peptidase | JHW38_15740 | QQP94698.1 | QQQ01576.1 | 86 | | WP_193830632.1 | | 94 | WP_057949581.1 | | 85 | WP_057920378.1 | | 69 | WP_082647636.1 | 53 |
| *secB* | protein-export chaperone SecB | JHW38_18100 | QQP95137.1 | QQQ01663.1 | 92 | | WP_074871611.1 | | 96 | WP_057949501.1 | | 92 | WP_036107609.1 | | 91 | WP_031372765.1 | 89 |
| *secA* | preprotein translocase subunit SecA | JHW38_11275 | QQP98517.1 | QQQ02449.1 | 97 | | WP_096376935.1 | | 97 | WP_057948802.1 | | 97 | WP_057922451.1 | | 93 | WP_057916944.1 | 91 |
| *secE* | preprotein translocase subunit SecE | JHW38_09795 | QQP98257.1 | QQQ02682.1 | 93 | | WP_074873738.1 | | 95 | WP_057948567.1 | | 93 | WP_036115412.1 | | 87 | WP_057918904.1 | 84 |
| *secY* | preprotein translocase subunit SecY | JHW38_09630 | QQP98226.1 | QQQ02714.1 | 100 | | WP_074872155.1 | | 99 | WP_057948537.1 | | 100 | WP_036111756.1 | | 95 | WP_031373816.1 | 96 |
| *ftsY* | signal recognition particle-docking protein FtsY | JHW38_08515 | QQP98031.1 | QQQ02920.1 | 74 | | WP_096377302.1 | | 97 | WP_057948362.1 | | 74 | WP_057921172.1 | | 91 | WP_057917394.1 | 74 |
| *yajC* | preprotein translocase subunit YajC | JHW38_08425 | QQP98013.1 | QQQ02938.1 | 84 | | WP_074865736.1 | | 95 | WP_057948347.1 | | 84 | WP_036101993.1 | | 87 | WP_031372943.1 | 81 |
| *ffh* | signal recognition particle protein | JHW38_07155 | QQP97781.1 | QQQ03137.1 | 97 | | WP_096377478.1 | | 99 | WP_057948157.1 | | 97 | WP_057921281.1 | | 95 | WP_057917550.1 | 95 |
| *yidC* | membrane protein insertase YidC | JHW38_16585 | QQP94856.1 | QQQ01404.1 | 97 | | WP_096382816.1 | | 91 | WP_057949735.1 | | 98 | WP_057923352.1 | | 81 | WP_057919817.1 | 81 |

NA = not available.
