# Supplementary material for: Comparative genomics provides insights into the potential biocontrol mechanism of two Lysobacter enzymogenes strains with distinct antagonistic activities
Source: Front Microbiol. 2022 Aug 11;13:966986. doi: 10.3389/fmicb.2022.966986 (PMC9410377; doi:10.3389/fmicb.2022.966986)
Supplement: Supplementary file 7 [file Table_9.DOCX]

**Supplementary Table 9** Homology analysis of selenium metabolism in *Lysobacter enzymogenes* CX03, CX06 and other representative *Lysobacter* strains.

| **Strain** |  | ***L. enzymogenes* CX03** | | ***L. enzymogenes* CX06** | | ***L. enzymogenes* M497-1** | | ***L. enzymogenes* C3** | | ***L. capsici* 55** | | ***L. antibioticus* 76** | | | |
| --- | --- | --- | --- | --- | --- | --- | --- | --- | --- | --- | --- | --- | --- | --- | --- |
| **Genes** | **Product Definition** | **Locus Tag** | **Protein ID** | **Protein ID** | **Homology (%)** | **Protein ID** | **Homology (%)** | **Protein ID** | **Homology (%)** | **Protein ID** | **Homology (%)** | | **Protein ID** | **Homology (%)** |  |
| **selenium association** | | | | | | | | | | | | | | |  |
| *mdeA* | cystathionine gamma-synthase | JHW38_11845 | QQP98623.1 | QQQ02344.1 | 97 | WP_096376851.1 | 97 | WP_057948903.1 | 96 | WP_057922519.1 | 94 | WP_057916859.1 | | 93 |  |
| *metE* | 5-methyltetrahydropteroyltriglutamate--homocysteine S-methyltransferase | JHW38_21400 | QQP95752.1 | QQQ00322.1 | 95 | WP_096379999.1 | 94 | WP_057949970.1 | 95 | WP_057920815.1 | 87 | WP_057920187.1 | | 86 |  |
| *metG* | methionine--tRNA ligase | JHW38_06785 | QQP97714.1 | QQQ03211.1 | 90 | WP_096377548.1 | 92 | WP_057948088.1 | 90 | WP_057921323.1 | 84 | WP_057917590.1 | | 83 |  |
| *metH* | methionine synthase | JHW38_04520 | QQP97312.1 | QQQ03592.1 | 92 | WP_096377954.1 | 93 | WP_057947743.1 | 92 | WP_057921730.1 | 87 | WP_057917894.1 | | 89 |  |
| *metH* | homocysteine S-methyltransferase family protein | JHW38_04515 | QQP97311.1 | QQQ03593.1 | 95 | WP_096377957.1 | 94 | WP_057947742.1 | 95 | WP_057921729.1 | 85 | WP_057917895.1 | | 85 |  |
| *trxB* | thioredoxin-disulfide reductase | JHW38_02970 | QQP97034.1 | QQP99196.1 | 98 | WP_096378727.1 | 98 | WP_057947444.1 | 98 | WP_036102909.1 | 93 | WP_057918101.1 | | 92 |  |
| *metC* | cystathionine beta-lyase | JHW38_19805 | QQP95462.1 | QQQ00605.1 | 92 | WP_198419989.1 | 93 | WP_197414813.1 | 92 | NA | NA | NA | | NA |  |
| *metB* | O-succinylhomoserine (thiol)-lyase | JHW38_22205 | QQP95897.1 | QQQ00194.1 | 94 | WP_096379683.1 | 96 | WP_057946539.1 | 94 | WP_082648346.1 | 94 | WP_082647743.1 | | 96 |  |
| **selenium transportation** | | | | | | | | | | | | | | |  |
| *cysN* | sulfate adenylyltransferase subunit CysN | JHW38_22730 | QQP95996.1 | QQQ00104.1 | 92 | WP_096379537.1 | 94 | WP_057946619.1 | 92 | WP_057920966.1 | 89 | WP_082648141.1 | | 86 |  |
| *cysD* | sulfate adenylyltransferase subunit CysD | JHW38_22725 | QQP95995.1 | QQQ00105.1 | 94 | WP_096379539.1 | 94 | WP_078996810.1 | 94 | WP_057923461.1 | 89 | WP_082647751.1 | | 93 |  |

NA= not available.
