# Supplementary material for: Comparative genomics provides insights into the potential biocontrol mechanism of two Lysobacter enzymogenes strains with distinct antagonistic activities
Source: Front Microbiol. 2022 Aug 11;13:966986. doi: 10.3389/fmicb.2022.966986 (PMC9410377; doi:10.3389/fmicb.2022.966986)
Supplement: Supplementary file 8 [file Table_10.DOCX]

**Supplementary Table 10** Homology analysis of biosynthesis of siderophore group non-ribosomal peptides in *Lysobacter enzymogenes* CX03 and other *Lysobacter* strains.

| **Strain** |  | ***L. enzymogenes* CX03** | | ***L. enzymogenes* CX06** | | ***L. enzymogenes* M497-1** | | ***L. enzymogenes* C3** | | ***L. capsici* 55** | | ***L. antibioticus* 76** | | | |
| --- | --- | --- | --- | --- | --- | --- | --- | --- | --- | --- | --- | --- | --- | --- | --- |
| **Genes** | **Product Definition** | **Locus Tag** | **Protein ID** | **Protein ID** | **Homology (%)** | **Protein ID** | **Homology (%)** | **Protein ID** | **Homology (%)** | **Protein ID** | **Homology (%)** | | **Protein ID** | **Homology (%)** |  |
| **Phenylalanine, tyrosine and tryptophan biosynthesis** | | | | | | | | | | | | | | | |
| *entA* | 2,3-dihydro-2,3-dihydroxybenzoate dehydrogenase | JHW38_03975 | QQP97215.1 | QQQ03661.1 | 80 | WP_096378132.1 | 90 | WP_057947682.1 | 81 | WP_057921683.1 | 70 | WP_057917951.1 | | 75 |  |
| *entC* | isochorismate synthase | JHW38_04000 | QQP97220.1 | QQQ03656.1 | 90 | WP_198420052.1 | 86 | WP_082644484.1 | 86 | WP_082648520.1 | 77 | WP_082647847.1 | | 78 |  |
| *entD* | 4'-phosphopantetheinyl transferase superfamily protein | JHW38_04325 | QQP97283.1 | QQQ01326.1 | 46 | WP_096376286.1 | 48 | WP_057949810.1 | 47 | WP_057923304.1 | 44 | WP_057919757.1 | | 47 |  |
| *entF* | enterochelin sythetase component F | JHW38_03985 | QQP97217.1 | QQQ03659.1 | 88 | WP_232518524.1 | 87 | WP_057947684.1 | 87 | WP_057921685.1 | 71 | LA76x_2596 | | 69 |  |
| *entE* | (2,3-dihydroxybenzoyl)adenylate synthase | JHW38_03995 | QQP97219.1 | QQQ03657.1 | 85 | WP_096378120.1 | 86 | WP_057947685.1 | 84 | WP_057921686.1 | 76 | WP_057917948.1 | | 77 |  |
| *dhbB* | isochorismatase family protein | JHW38_03990 | QQP97218.1 | QQQ03658.1 | 93 | WP_096378123.1 | 92 | WP_175429202.1 | 93 | WP_187313392.1 | 83 | WP_057918506.1 | | 83 |  |
| **Nonribosomal peptide structures** | | | | | | | | | | | | | | |  |
| *mcyF* | aspartate/glutamate racemase family protein | JHW38_16245 | QQP94790.1 | QQQ01465.1 | 86 | WP_096376202.1 | 93 | WP_197414793.1 | 86 | WP_036103416.1 | 81 | WP_057919825.1 | | 86 |  |
| *racD* | amino acid racemase | JHW38_21820 | QQP95826.1 | QQQ00256.1 | 92 | WP_096379799.1 | 92 | WP_057946486.1 | 92 | WP_057920860.1 | 83 | WP_148649914.1 | | 79 |  |
