# Supplementary material for: Comparative genomics provides insights into the potential biocontrol mechanism of two Lysobacter enzymogenes strains with distinct antagonistic activities
Source: Front Microbiol. 2022 Aug 11;13:966986. doi: 10.3389/fmicb.2022.966986 (PMC9410377; doi:10.3389/fmicb.2022.966986)
Supplement: Supplementary file 9 [file Table_11.DOCX]

**Supplementary Table 11** Homology analysis of genes related to polyketone, lipopolysaccharide, peptidoglycan biosynthesis in *Lysobacter enzymogenes* CX03, CX06 and other representative *Lysobacter* strains.

| **Strain** |  | ***L. enzymogenes* CX03** | | ***L. enzymogenes* CX06** | | ***L. enzymogenes* M497-1** | | ***L. enzymogenes* C3** | | ***L. capsici* 55** | | ***L. antibioticus* 76** | | | |
| --- | --- | --- | --- | --- | --- | --- | --- | --- | --- | --- | --- | --- | --- | --- | --- |
| **Genes** | **Product Definition** | **Locus Tag** | **Protein ID** | **Protein ID** | **Homology (%)** | **Protein ID** | **Homology (%)** | **Protein ID** | **Homology (%)** | **Protein ID** | **Homology (%)** | | **Protein ID** | **Homology (%)** |  |
| **Polyketide sugar unit biosynthesis** | | | | | | | | | | | | | | | |
| *rfbA* | glucose-1-phosphate thymidylyltransferase RfbA | JHW38_09075 | QQP98125.1 | QQQ02820.1 | 97 | WP_096377221.1 | 98 | WP_057948438.1 | 97 | WP_036101814.1 | 88 | WP_057917330.1 | | 91 |  |
| *rfbB* | dTDP-glucose 4,6-dehydratase | JHW38_09080 | QQP98126.1 | QQQ02819.1 | 94 | WP_096377220.1 | 96 | WP_057948439.1 | 95 | WP_057921075.1 | 89 | WP_057917329.1 | | 87 |  |
| *rfbC* | dTDP-4-dehydrorhamnose 3,5-epimerase | JHW38_09070 | QQP98124.1 | QQQ02821.1 | 81 | WP_096377222.1 | 87 | WP_057948437.1 | 81 | WP_057921076.1 | 81 | WP_057917331.1 | | 79 |  |
| *rfbD* | dTDP-4-dehydrorhamnose reductase | JHW38_09065 | QQP98837.1 | QQQ02822.1 | 84 | WP_096383206.1 | 85 | WP_057948436.1 | 83 | WP_057921077.1 | 69 | WP_057917332.1 | | 71 |  |
| **Lipopolysaccharide biosynthesis** | | | | | | | | | | | | | | | |
| *lpxA* | acyl-ACP--UDP-N-acetylglucosamine acyltransferase | JHW38_07655 | QQP97877.1 | QQQ03041.1 | 93 | WP_074866119.1 | 94 | WP_057948255.1 | 93 | WP_057921235.1 | 81 | WP_057917475.1 | | 84 |  |
| *lpxB* | lipid-A-disaccharide synthase | JHW38_07665 | QQP97879.1 | QQQ03039.1 | 95 | WP_096377395.1 | 94 | WP_082644553.1 | 94 | WP_082648412.1 | 90 | WP_082647782.1 | | 91 |  |
| *lpxC* | UDP-3-O-acyl-N-acetylglucosamine deacetylase | JHW38_11290 | QQP98520.1 | QQQ02446.1 | 97 | WP_096376933.1 | 97 | WP_057948805.1 | 97 | WP_036104603.1 | 93 | WP_057916942.1 | | 95 |  |
| *lpxD* | UDP-3-O-(3-hydroxymyristoyl)glucosamine N-acyltransferase | JHW38_07645 | QQP97875.1 | QQQ03043.1 | 93 | WP_096377397.1 | 92 | WP_057948253.1 | 91 | WP_057921236.1 | 89 | WP_057917476.1 | | 86 |  |
| *lpxH* | UDP-2,3-diacylglucosamine diphosphatase | JHW38_23185 | QQP96081.1 | QQQ00013.1 | 95 | WP_096379430.1 | 95 | WP_057946705.1 | 96 | WP_057922263.1 | 89 | WP_057918846.1 | | 87 |  |
| *lpxK* | tetraacyldisaccharide 4'-kinase | JHW38_06220 | QQP97608.1 | QQQ03314.1 | 92 | WP_096377632.1 | 93 | WP_057947993.1 | 92 | WP_057921389.1 | 85 | WP_057917685.1 | | 83 |  |
| *lpxL* | LpxL/LpxP family Kdo(2)-lipid IV(A) lauroyl/palmitoleoyl acyltransferase | JHW38_20690 | QQP95620.1 | QQQ00456.1 | 95 | WP_096380497.1 | 97 | WP_057946307.1 | 95 | WP_057920731.1 | 93 | WP_057920198.1 | | 90 |  |
| *htrB* | lauroyl acyltransferase | JHW38_18895 | QQP95287.1 | QQQ01022.1 | 93 | WP_096381561.1 | 93 | WP_057945981.1 | 93 | WP_057922832.1 | 87 | WP_057919857.1 | | 86 |  |
| *lpxO* | lipid A hydroxylase LpxO | JHW38_11465 | QQP98551.1 | QQQ02414.1 | 96 | WP_074863295.1 | 99 | WP_082644641.1 | 96 | WP_036104706.1 | 90 | WP_031371572.1 | | 87 |  |
| *lpxR* | lipid A deacylase LpxR family protein | JHW38_13430 | QQP94275.1 | QQQ02052.1 | 95 | WP_096376613.1 | 97 | WP_057949163.1 | 95 | WP_057922716.1 | 84 | WP_057916644.1 | | 82 |  |
| *waaA* | lipid IV(A) 3-deoxy-D-manno-octulosonic acid transferase | JHW38_20695 | QQP95621.1 | QQQ00455.1 | 96 | WP_096380494.1 | 96 | WP_057946308.1 | 96 | WP_036111287.1 | 93 | WP_057919130.1 | | 93 |  |
| *kdkA* | 3-deoxy-D-manno-octulosonic acid kinase | JHW38_24640 | QQP96349.1 | QQP99748.1 | 93 | WP_096379174.1 | 93 | WP_057946959.1 | 93 | WP_046657246.1 | 86 | WP_057918636.1 | | 85 |  |
| *eptA* | phosphoethanolamine--lipid A transferase | JHW38_18615 | QQP95232.1 | QQQ01778.1 | 89 | WP_096381760.1 | 92 | WP_057949401.1 | 89 | WP_057922894.1 | 80 | WP_057916276.1 | | 82 |  |
| *kdsA* | 3-deoxy-8-phosphooctulonate synthase | JHW38_23790 | QQP96190.1 | QQP99902.1 | 99 | WP_096379301.1 | 97 | WP_057946814.1 | 99 | WP_046657456.1 | 97 | WP_031374364.1 | | 97 |  |
| *kdsB* | 3-deoxy-manno-octulosonate cytidylyltransferase | JHW38_06215 | QQP97607.1 | QQQ03315.1 | 96 | WP_096377633.1 | 96 | WP_057947992.1 | 97 | WP_057921390.1 | 89 | WP_057917686.1 | | 89 |  |
| *kdsC* | HAD hydrolase family protein | JHW38_22555 | QQP95962.1 | QQQ00137.1 | 96 | WP_096379587.1 | 96 | WP_057946590.1 | 97 | WP_036102291.1 | 91 | WP_057917245.1 | | 85 |  |
| *kdsD* | KpsF/GutQ family sugar-phosphate isomerase | JHW38_22560 | QQP95963.1 | QQQ00136.1 | 93 | WP_096379585.1 | 96 | WP_057946591.1 | 93 | WP_036102732.1 | 92 | WP_057917246.1 | | 91 |  |
| **Peptidoglycan biosynthesis** | | | | | | | | | | | | | | | |
| *murA* | UDP-N-acetylglucosamine 1-carboxyvinyltransferase | JHW38_22570 | QQP95965.1 | QQQ00134.1 | 95 | WP_096379583.1 | 97 | WP_057946593.1 | 96 | WP_057920945.1 | 94 | WP_057917248.1 | | 94 |  |
| *murB* | UDP-N-acetylmuramate dehydrogenase | JHW38_25080 | QQP96431.1 | QQP99673.1 | 90 | WP_096379116.1 | 92 | WP_057947028.1 | 90 | WP_057922024.1 | 82 | WP_057918573.1 | | 80 |  |
| *murC* | UDP-N-acetylmuramate--L-alanine ligase | JHW38_11315 | QQP98524.1 | QQQ03821.1 | 97 | WP_096383104.1 | 97 | WP_057950254.1 | 97 | WP_057922453.1 | 96 | WP_057916938.1 | | 95 |  |
| *murE* | UDP-N-acetylmuramoyl-L-alanyl-D-glutamate--2,6-diaminopimelate ligase | JHW38_11340 | QQP98868.1 | QQQ02437.1 | 97 | WP_172437358.1 | 96 | WP_175429384.1 | 97 | WP_057923647.1 | 91 | WP_057916933.1 | | 85 |  |
| *murF* | UDP-N-acetylmuramoyl-tripeptide--D-alanyl-D-alanine ligase | JHW38_11335 | QQP98528.1 | QQQ02438.1 | 91 | WP_096376929.1 | 90 | WP_057948813.1 | 90 | WP_057922456.1 | 78 | WP_057916934.1 | | 80 |  |
| *murG* | undecaprenyldiphospho-muramoylpentapeptide beta-N-acetylglucosaminyltransferase | JHW38_11320 | QQP98525.1 | QQQ02441.1 | 95 | WP_096376931.1 | 98 | WP_057948810.1 | 95 | WP_057922454.1 | 89 | WP_057916937.1 | | 86 |  |
| *murY* | phospho-N-acetylmuramoyl-pentapeptide-transferase | JHW38_11330 | QQP98527.1 | QQQ02439.1 | 98 | WP_074862625.1 | 98 | WP_057948812.1 | 98 | WP_036104620.1 | 89 | WP_057916935.1 | | 89 |  |
| *bacA* | undecaprenyl-diphosphate phosphatase | JHW38_15775 | QQP94704.1 | QQQ01570.1 | 95 | WP_096382658.1 | 95 | WP_057949586.1 | 95 | WP_057920376.1 | 92 | WP_057919506.1 | | 91 |  |
| *ddlA* | D-alanine--D-alanine ligase | JHW38_11310 | QQP98867.1 | QQQ02442.1 | 97 | WP_172437359.1 | 96 | WP_057948809.1 | 97 | WP_046657795.1 | 89 | WP_148649909.1 | | 89 |  |
| *bcrC* | phosphatase PAP2 family protein | JHW38_24510 | QQP96327.1 | QQP99773.1 | 97 | WP_096379191.1 | 98 | WP_057946935.1 | 98 | WP_057922096.1 | 92 | WP_057918653.1 | | 96 |  |
| *uppS* | di-trans,poly-cis-decaprenylcistransferase | JHW38_07620 | QQP97870.1 | QQQ03048.1 | 95 | WP_096377401.1 | 97 | WP_057948249.1 | 95 | WP_036113712.1 | 90 | WP_057917481.1 | | 86 |  |
| *mrcA* | penicillin-binding protein 1A | JHW38_21280 | QQP95728.1 | QQQ00349.1 | 92 | WP_096380078.1 | 96 | WP_057946403.1 | 92 | WP_057920792.1 | 86 | WP_057919052.1 | | 85 |  |
| *mrcB* | penicillin-binding protein 1B | JHW38_10625 | QQP98861.1 | QQQ02544.1 | 96 | WP_096377025.1 | 97 | WP_057948685.1 | 96 | WP_057922395.1 | 92 | WP_057920183.1 | | 88 |  |
| *pbpC* | penicillin-binding protein 1C | JHW38_01900 | QQP98740.1 | QQP99364.1 | 94 | WP_096378874.1 | 94 | WP_082644430.1 | 95 | WP_057921853.1 | 88 | WP_057918284.1 | | 87 |  |
| *mtgA* | monofunctional biosynthetic peptidoglycan transglycosylase | JHW38_22150 | QQP95886.1 | QQQ00206.1 | 85 | WP_083382373.1 | 96 | WP_057946532.1 | 85 | WP_057923448.1 | 86 | WP_057919954.1 | | 84 |  |
| *mrdA* | penicillin-binding protein 2 | JHW38_13085 | QQP94210.1 | NA | NA | WP_096376666.1 | 95 | WP_082644674.1 | 95 | WP_057922677.1 | 84 | WP_057916694.1 | | 81 |  |
| *ftsI* | penicillin-binding protein 2 | JHW38_11345 | QQP98869.1 | QQQ02436.1 | 93 | WP_096383100.1 | 95 | WP_082644637.1 | 93 | WP_082124471.1 | 90 | WP_082647702.1 | | 91 |  |
| *dacA* | D-alanyl-D-alanine carboxypeptidase | JHW38_12905 | QQP98885.1 | QQQ03805.1 | 97 | WP_074861845.1 | 98 | WP_057949088.1 | 97 | WP_082648701.1 | 91 | WP_082647669.1 | | 92 |  |
| *vanY* | D-alanyl-D-alanine carboxypeptidase family protein | JHW38_24265 | QQP96279.1 | QQQ03974.1 | 93 | WP_096379231.1 | 91 | WP_057946897.1 | 93 | WP_057922121.1 | 85 | WP_057918688.1 | | 82 |  |
| **Carbapenem biosynthesis** | | | | | | | | | | | | | |  | |
| *proB* | glutamate 5-kinase | JHW38_00670 | QQP96605.1 | QQP99602.1 | 94 | WP_096379799.1 | 93 | WP_057946486.1 | 93 | WP_082648564.1 | 85 | WP_057918460.1 | | 86 |  |
| *proA* | glutamate-5-semialdehyde dehydrogenase | JHW38_00675 | QQP96606.1 | QQP99601.1 | 90 | WP_096379053.1 | 91 | WP_057947090.1 | 90 | WP_057921981.1 | 85 | WP_057918459.1 | | 85 |  |
