# Supplementary figures and images for: Comparative genomics provides insights into the potential biocontrol mechanism of two Lysobacter enzymogenes strains with distinct antagonistic activities
Source: Front Microbiol. 2022 Aug 11;13:966986. doi: 10.3389/fmicb.2022.966986 (PMC9410377; doi:10.3389/fmicb.2022.966986)

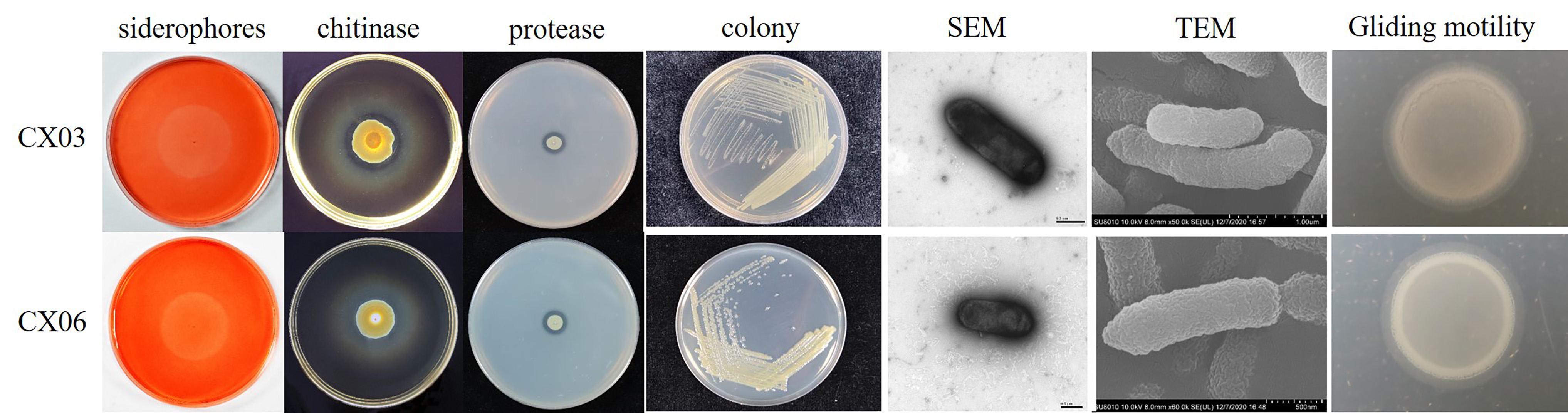

Supplement: Supplementary Figure 1 — Production of siderophores, extracellular enzymes and general morphological characteristics, gliding motility of L. enzymogenes CX03 and CX06. [file Image_1.JPEG]

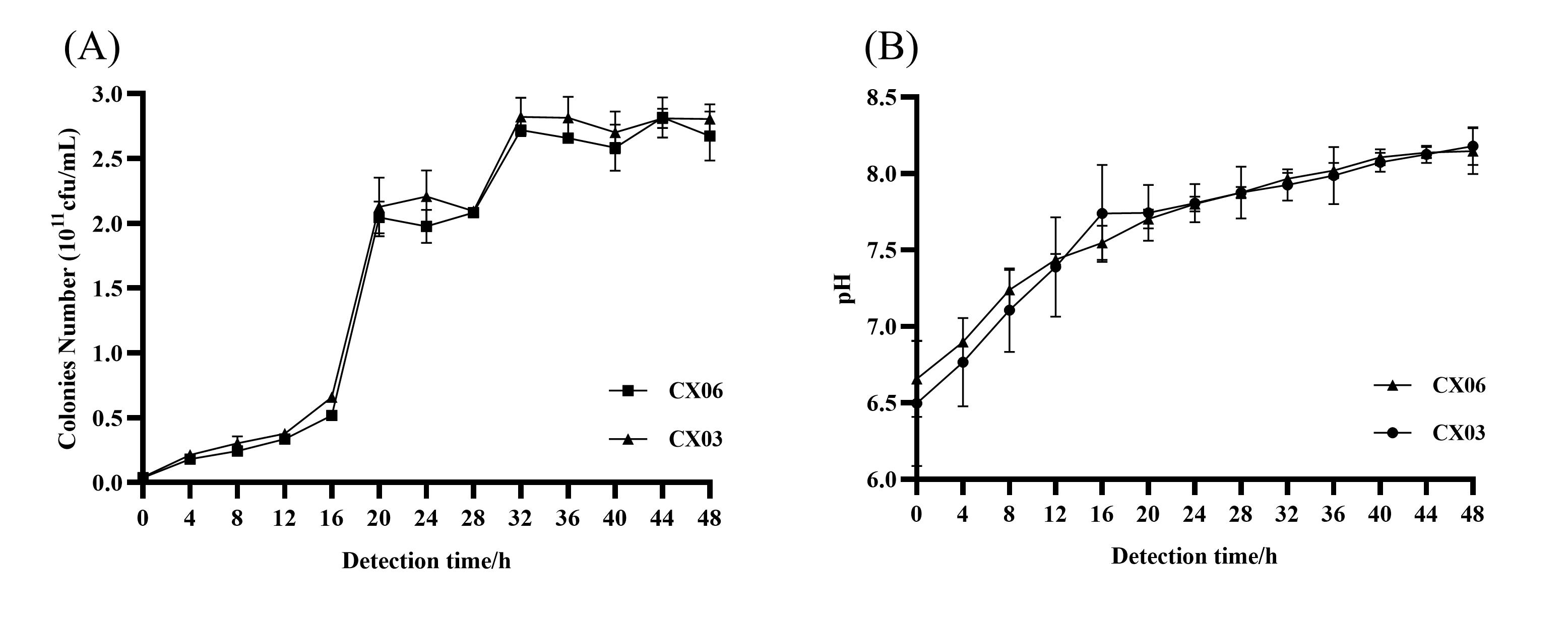

Supplement: Supplementary Figure 2 — Growth and pH curves of L. enzymogenes CX03 and CX06. (A) Colony concentrations of CX03 and CX06 at different culture times. (B) pH values of CX03 and CX06 suspensions at different culture times. [file Image_2.JPEG]

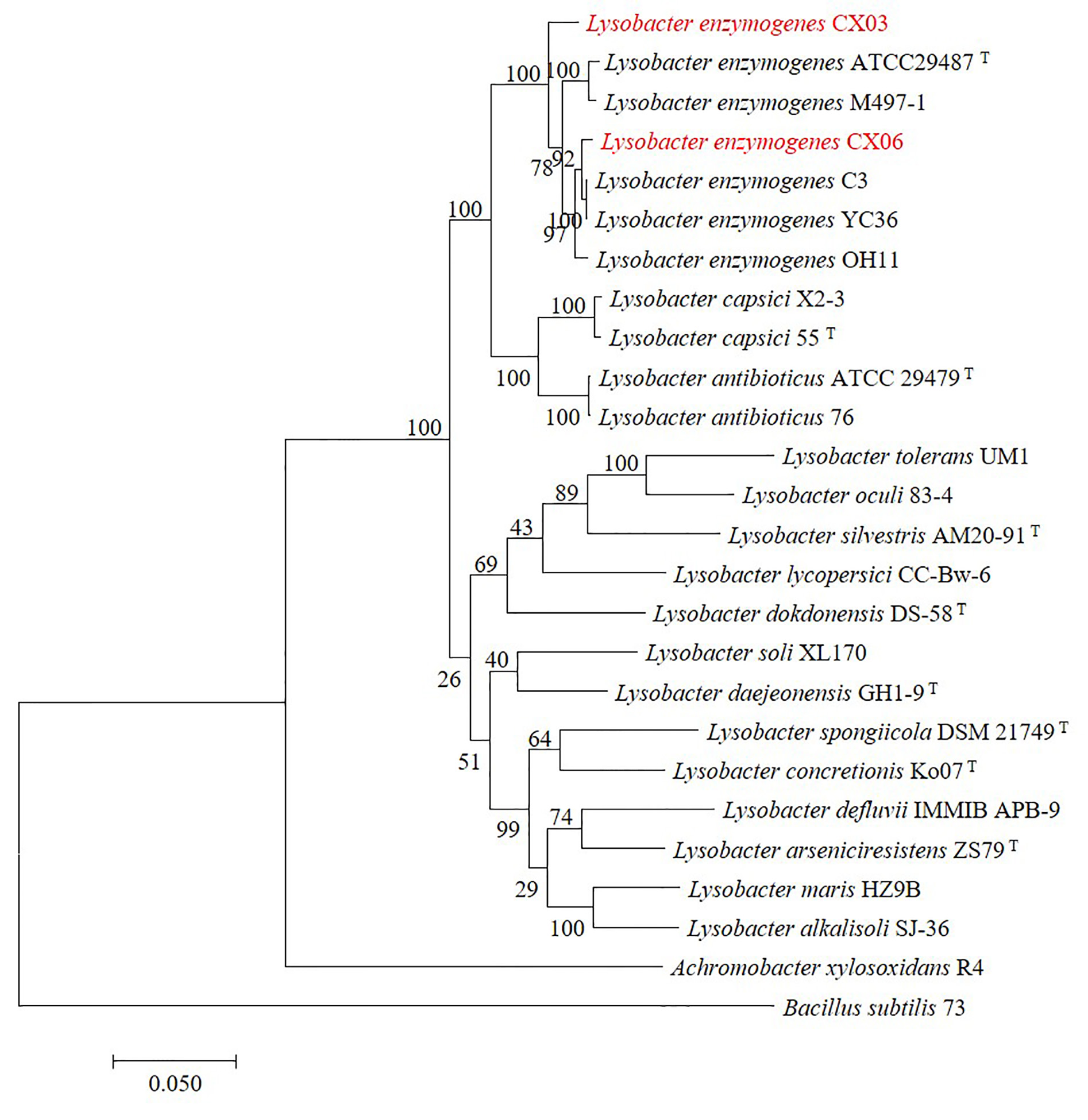

Supplement: Supplementary Figure 3 — Phylogenetic tree highlighting the relative positions of L. enzymogenes CX03 and CX06 among other Lysobacter strains. The phylogenetic tree was constructed based on four housekeeping genes (16S rRNA, gyrB, atpD, rpoD) according to the aligned gene sequences using maximum likelihoods derived from MEGA 6.0 software. Bootstrap values (1,000 replicates) were shown at the branch points. The scale bar indicates 0.05 nucleotide substitutions pernucleotide position. [file Image_3.JPEG]

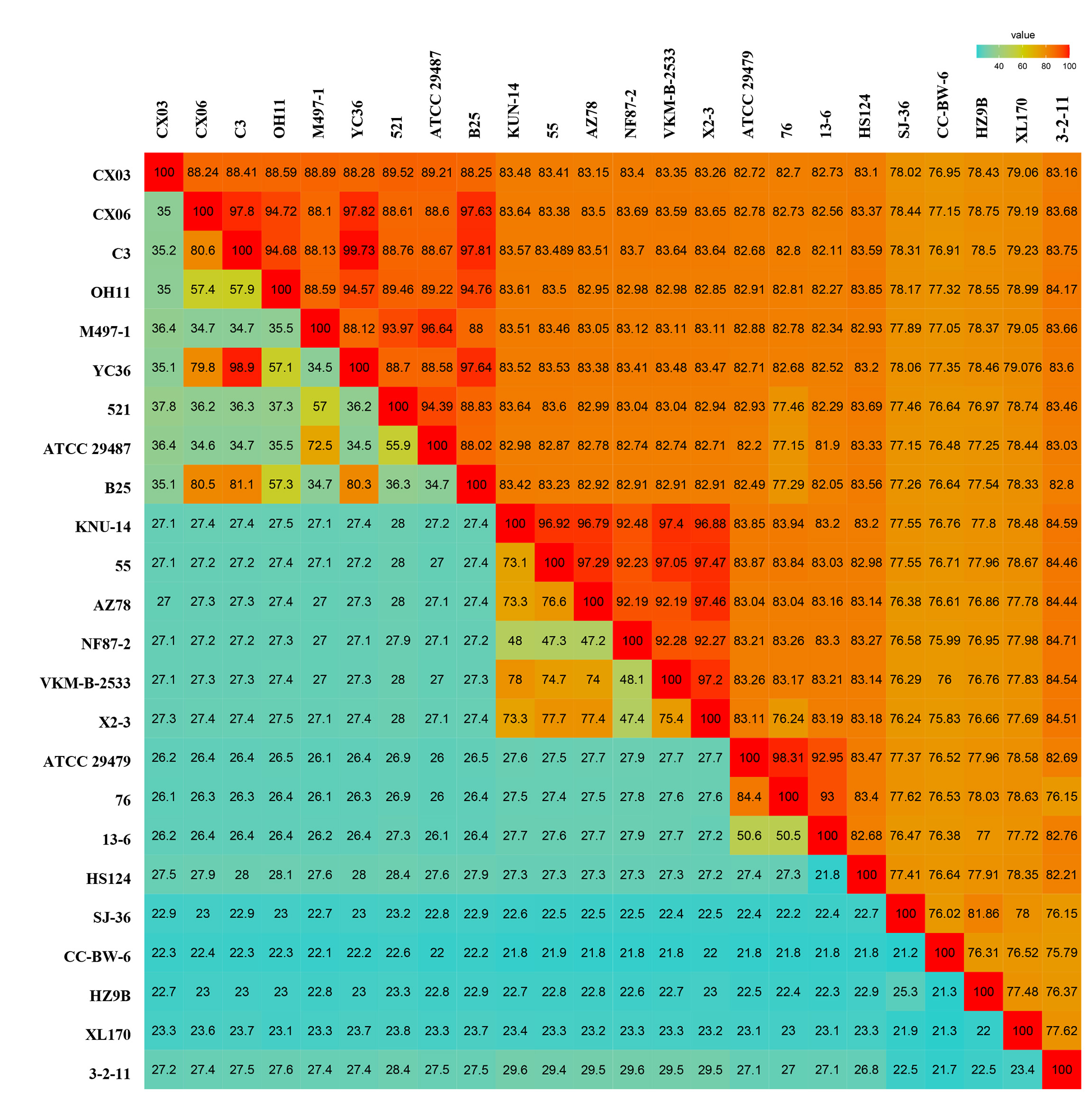

Supplement: Supplementary Figure 4 — Percentage of the average nucleotide identities (ANI) and in silico DNA-DNA hybridization (DDH) among the selected Lysobacter strains. ANI values were computed for a pairwise genome comparison using the OrthoANIu algorithm. The percentage of ANI was shown on the top right. DDH values were calculated by using the Genome-to-Genome Distance Calculator (GGDC). The percentage of DDH was shown on the bottom left. [file Image_4.JPEG]

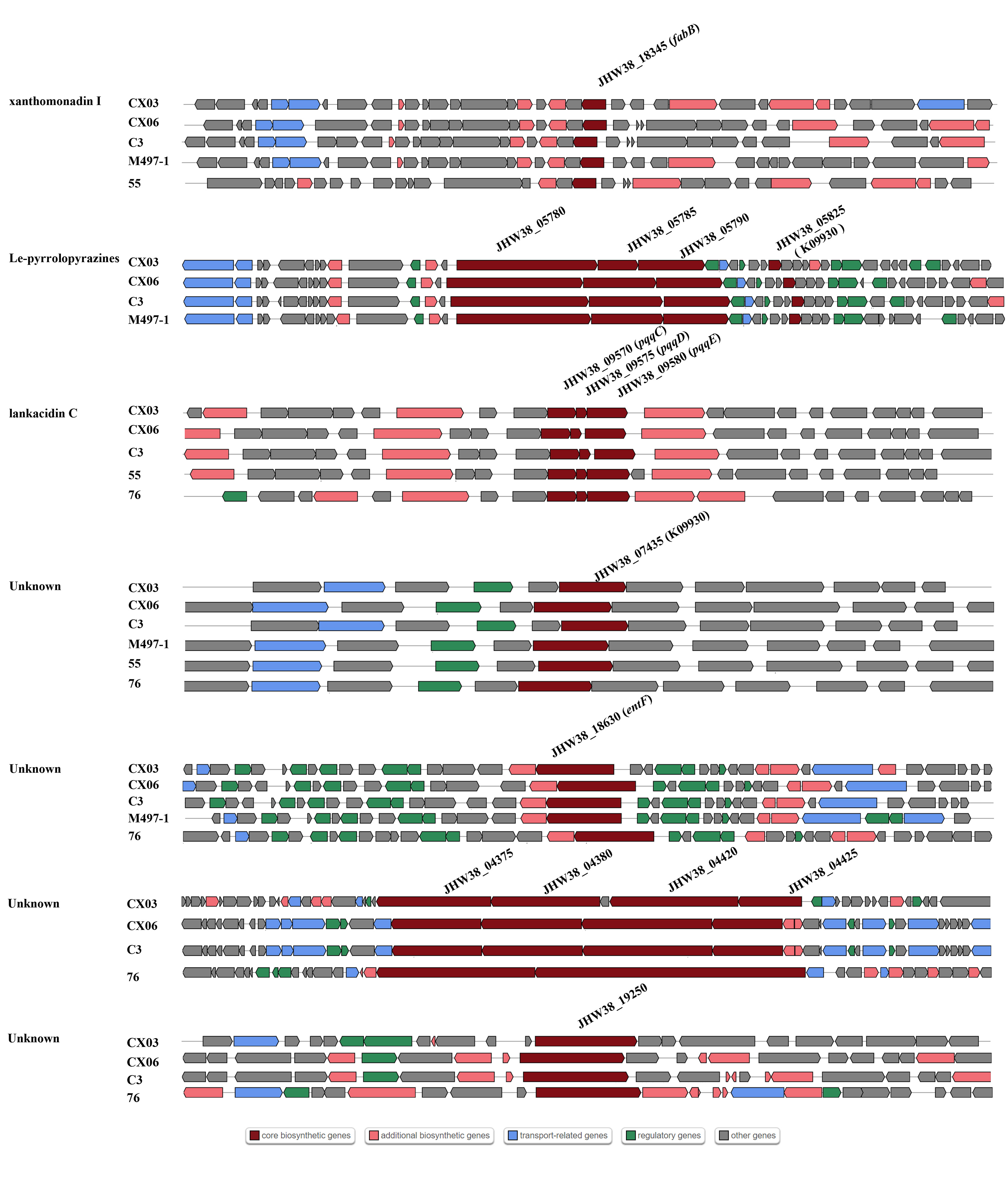

Supplement: Supplementary Figure 5 — Comparisons of gene clusters for secondary metabolites (both existed in CX03 and CX06) with other representative Lysobacter strains C3, M497-1, 55 and 76. Dark red indicated the core biosynthetic genes in different gene clusters. The core biosynthesis genes were marked in the diverse gene clusters. [file Image_5.JPEG]

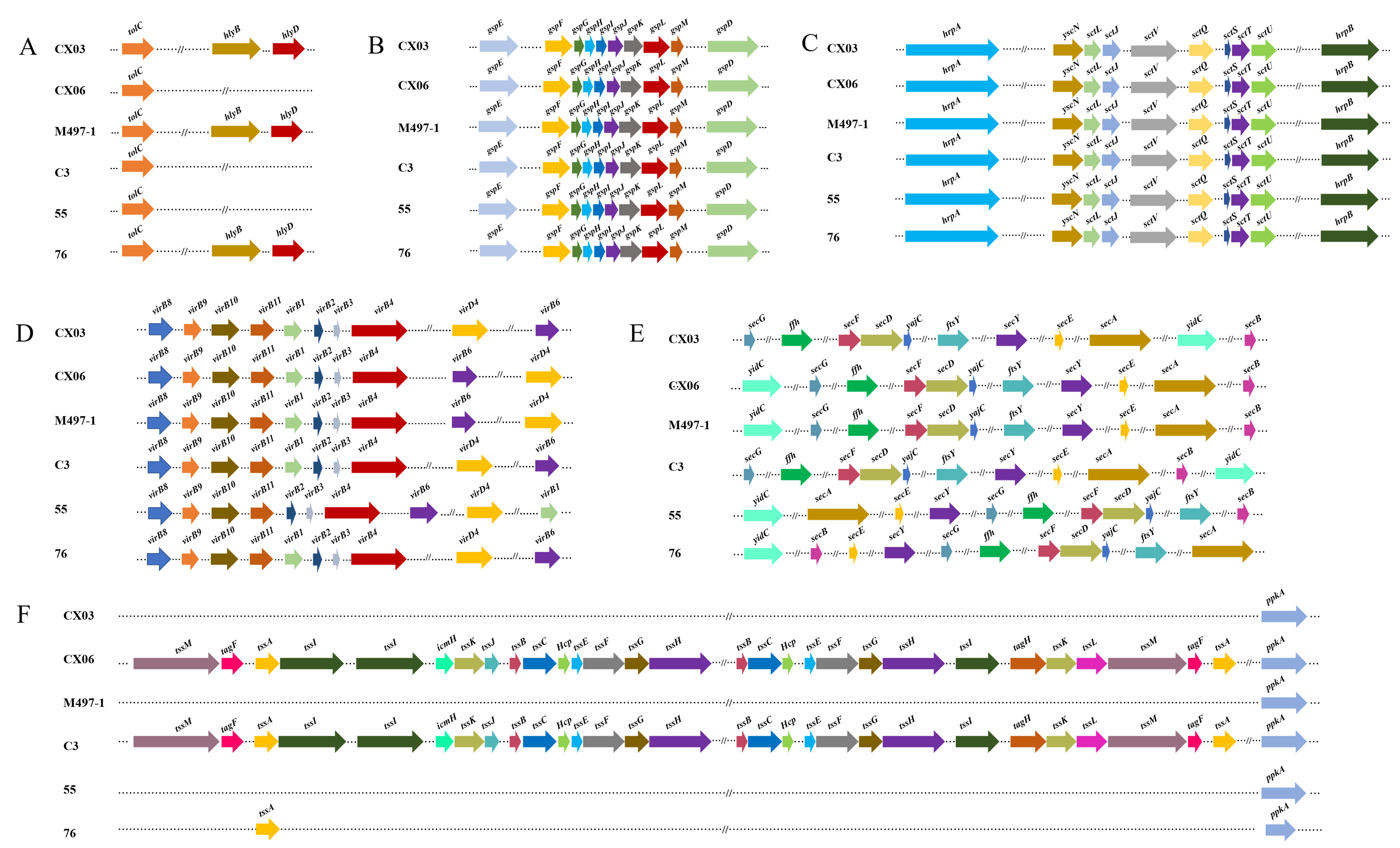

Supplement: Supplementary Figure 6 — Comparison of the bacterial secretion system gene clusters of L. enzymogenes CX03 and CX06 against four previous fully sequenced Lysobacter genomes. (A) Type I secretion system. (B) Type II secretion system. (C) Type III secretion system. (D) Type IV secretion system. (E) Sec (secretion) system. (F) Type VI secretion system. The same color represented genes with the same or similar biological function. Arrows denoted putative transcriptional units. The length of blocks represented the size of genes (1 cm = 1000 bp). [file Image_6.JPEG]

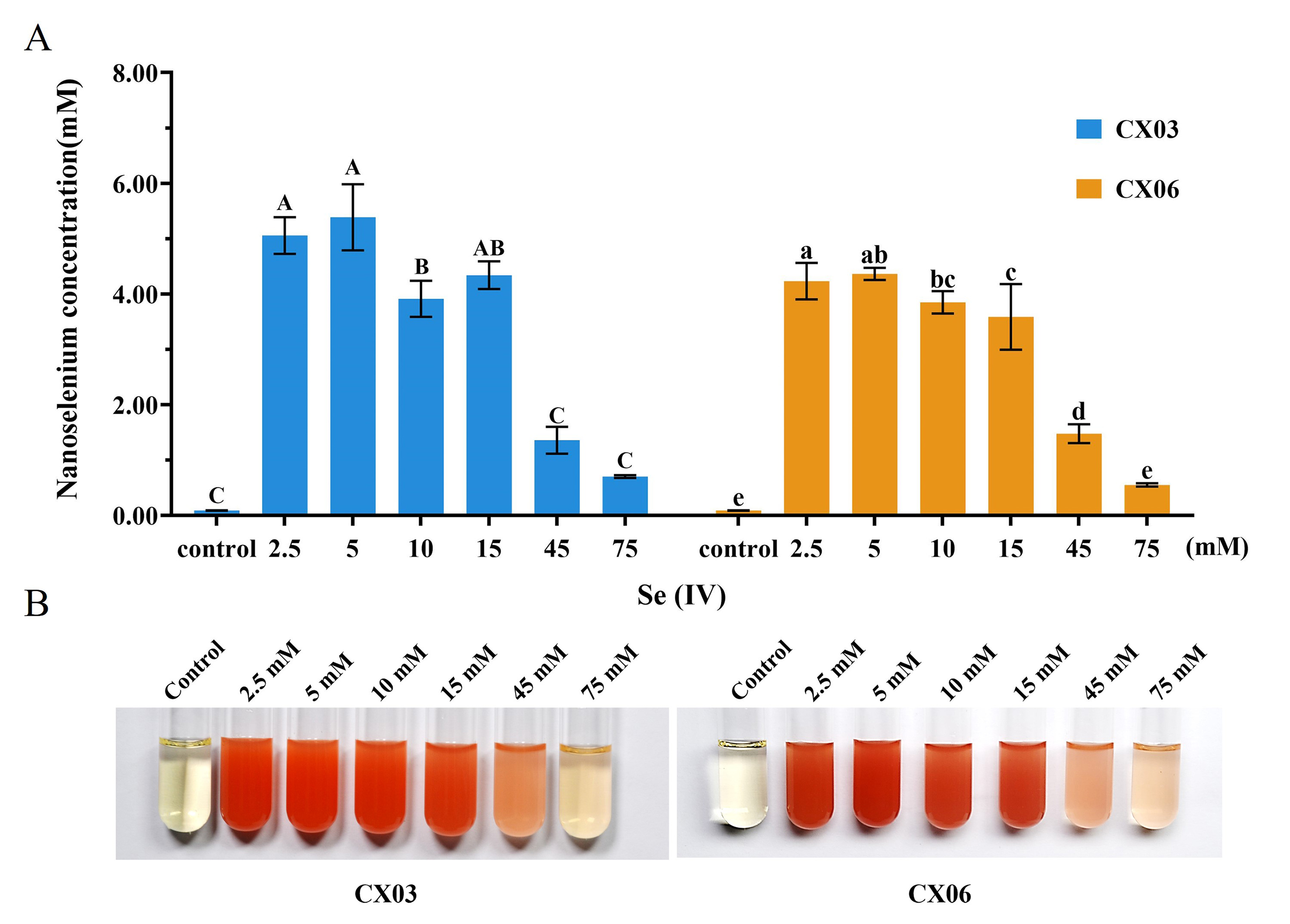

Supplement: Supplementary Figure 7 — Determination of selenium synthesis capabilities of L. enzymogenes CX03 and CX06. (A) Nanoselenium formation by strains CX03 and CX06 grown in LB broth supplemented with different concentrations of Na2SO3. Error bars represent the range of standard errors. Three biological replicates were performed for each treatment, and the experiments were independently repeated three times. (B) The survival of strains CX03 and CX06 in different concentrations of Na2SO3. [file Image_7.JPEG]
